# Photoswitchable imines: aryliminopyrazoles quantitatively convert to long-lived *Z*-isomers with visible light†

**DOI:** 10.1039/d3sc05841g

**Published:** 2024-02-14

**Authors:** Jiarong Wu, Lasse Kreimendahl, Suyuan Tao, Olga Anhalt, Jake L. Greenfield

**Affiliations:** a Institut für Organische Chemie, Universität Würzburg 97074 Würzburg Germany Jake.Greenfield@uni-wuerzburg.de; b Center for Nanosystems Chemistry (CNC), Universität Würzburg 97074 Würzburg Germany

## Abstract

Arylimines offer promise in dynamic-covalent materials due to their recyclability and ease of synthesis. However, their light-triggered *E*/*Z* isomerism has received little attention. This is attributed to challenges that include low thermal stability of their metastable state (<60 s at 20 °C), incomplete photoswitching (<50% to the metastable state), and the need for UV light (≤365 nm). We overcome these limitations with a novel class of imine photoswitch, the aryliminopyrazoles (AIPs). These AIPs can be switched using visible light (470 nm), attain photostationary states with over 95% of the *Z*-isomer, exhibit great resistance to fatigue, and have thermal half-lives up to 19.2 hours at room temperature. Additionally, they display T-type and negative photochromism under visible light irradiation—a useful property. The photochromic properties, quantitative assembly and accessibility of precursors set these photoswitches apart from their azo-based analogues. These findings open avenues for next-generation photoresponsive dynamic-covalent materials driven solely by these new photochromic linkages and further exploration of photocontrolled dynamic combinatorial chemistry.

## Abstract 2

Simple structural modifications significantly boost the photochromic performance of imine-based photoswitches. This work lays a foundation for exploring new motifs in light-addressable dynamic combinatorial chemistry.

## Graphical abstract

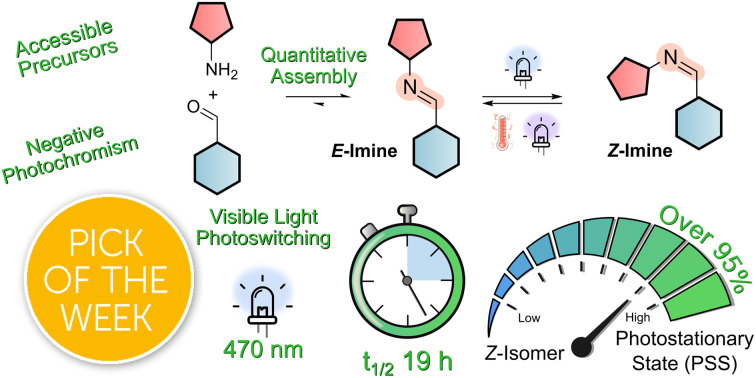

## Introduction

Molecular photoswitches, molecules that can reversibly switch between two or more states in response to light, are frequently used in the construction of light-responsive materials^1–8^ and the operation of functional molecular machines.^9–13^ In nature, the photoinduced *E*/*Z* isomerisation of a *cis* C=C bond in retinal to the *trans* conformation is fundamental in the mechanism of vision, whilst commercial applications of photoswitches include photochromic lenses, energy storage materials,^14,15^ and volumetric 3D printers.^16^ Of the various classes of photoswitches,^17,18^ those that display a photoinduced *trans*–*cis* (*E*/*Z*) isomerism are arguably the most commonly studied.^3,19–24^ Examples of such switches include derivatives of stilbene (C=C),^25,26^ azobenzene and diazocine (N=N),^19–21,27,28^ and subsets of imines (C=N) that include hydrazones^29^ and acylhydrazones.^22,30^ Imines, specifically sterically hindered benzil-derived imines,^31^ ketimines^32–34^ and iminothioindoxyls^3,35^ have shown promise as photoswitches and light-activated molecular motors that display unidirectional rotation about the imine bond.^12^

**Fig. 1:**
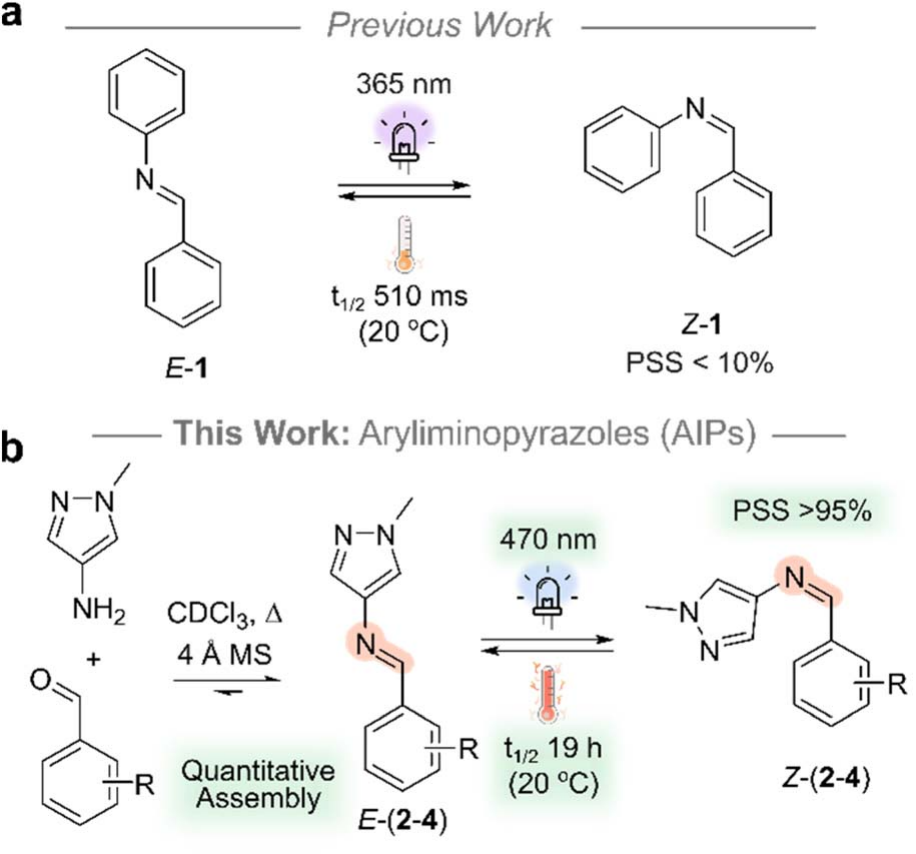
(a) Diphenylimine, 1, used as a reference compound for comparison. (b) AIPs derived from aminopyrazole and benzaldehyde precursors. Experimental details are provided in the ESI.†

To date, photoswitches derived from arylimines have been seldomly explored despite their straightforward preparation and structural similarity to azobenzene and stilbene.^30,36^ This is attributed to their poor photoswitching properties, rendering them challenging systems to study (Fig. 1a). These properties include: (i) short thermal half-lives (*t*_1/2_)^37,38^ that typically range from 10^−3^ to 10^1^ s at room temperature (Table S3†); (ii) low completeness of photoswitching to the metastable state at the photostationary state (PSS, <50%, Table S3†); (iii) the need for high energy UV light to induce photoisomerism, typically in the range of 254 to 365 nm.^30^ Overcoming these limitations would expand the toolkit of available photochromic compounds and enable the creation of new dynamic-covalent materials^39,40^ that responds to light directly at the imine bond.^41^ Such materials would be amenable to build photoresponsive polymers,^42^ 2D and 3D architectures^43^ and act as more accessible azo-analogues in photopharmacology.^44,45^

In the context of azo-based photoswitches, several strategies to enhance photoswitching properties have been identified and implemented in light-responsive applications. The substitution of one,^46^ or both,^47,48^ of the arene rings with a heteroarene has proved to be a reliable strategy for tuning the photoswitchable properties.^17,49^ Of the azoheteroarenes, the arylazopyrazoles are one of the most studied classes and have displayed quantitative bidirectional switching,^46,50,51^ as well as *t*_1/2_ reaching tens of years.^52–54^ Moreover, the *ortho*-amination of the phenyl rings in azobenzene-based^55,56^ and arylazopyrazole^57^ photoswitches achieved a bathochromic shift of the π–π* electronic absorption band with a concomitant reduction in *t*_1/2_.^55,56^ Inspired by these recent advancements in azo-based photoswitches and the timeliness of light-controlled systems, we turned our attention to the relatively over-looked imine photoswitches.

In this work, we address the previously reported poor performance of the arylimine photoswitches: aryliminopyrazoles (AIPs) are introduced as an accessible class of photoswitch which exhibits significantly enhanced photoswitching properties compared to their non-heteroaryl analogues (Fig. 1). Specifically, we explore the impact of *ortho*-amination—both mono- and di-amination—of the phenyl ring with pyrrolidine on the photoswitching properties. We demonstrate the quantitative assembly of AIP photoswitches that can be switched with visible light (470 nm), achieve photostationary states in excess of 95% of the *Z*-isomer, and exhibit *t*_1/2_ of the *Z*-isomer that reach 19.2 hours at room temperature. Importantly, the AIPs can be assembled quantitatively, and the accessibility of precursors renders this class of photoswitch amenable to large scale application.

## Results and discussion

**Fig. 2:**
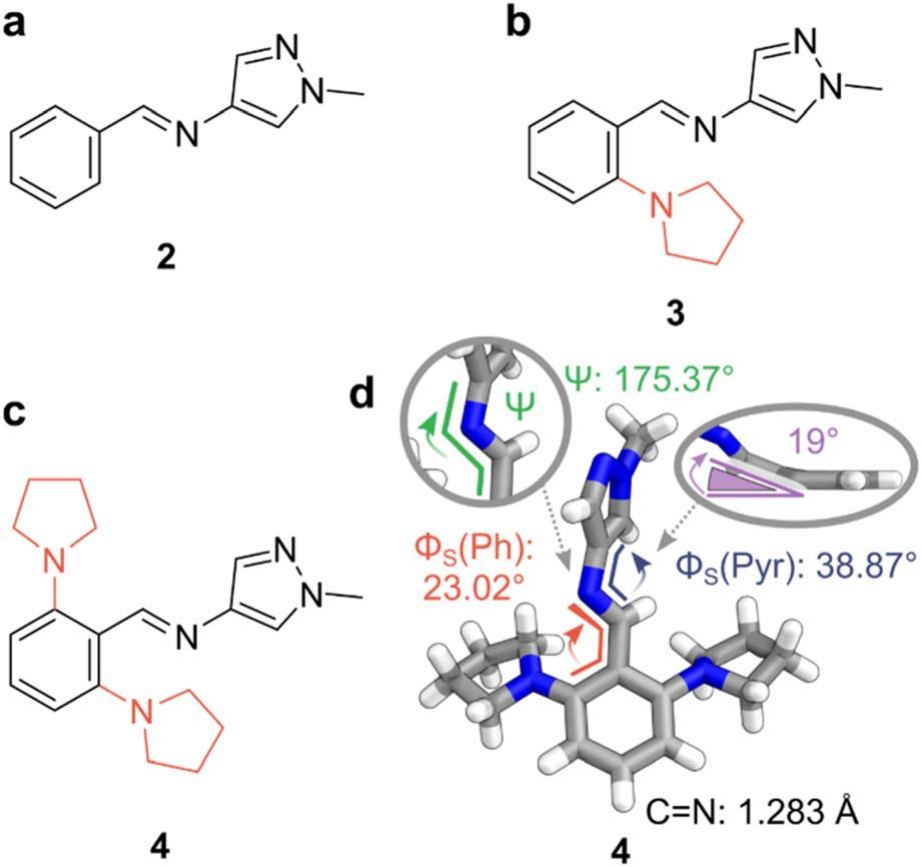
Photoswitchable AIPs and X-ray crystal structure. (a) Replacement of a phenyl ring with a pyrazole unit. (b) Substitution of AIP 2 with a pyrrolidine at the *ortho*-position furnished photoswitch 3. (c) Substitution at both *ortho*-positions of the phenyl ring with pyrrolidine yields AIP 4. (d) The X-ray crystal structure of 4. (inset, right) Deviation of the C=N bond from the plane containing the phenyl ring, with pyrrolidine units hidden for clarity. Color code: C, grey; N, blue; H, white. CCDC 2293811.

To quantitatively prepare the AIPs in Fig. 2, the corresponding aldehyde and amine precursors were combined in CDCl_3_ and heated to reflux overnight with 4 Å molecular sieves (MS). Trace amounts of DCl in CDCl_3_ were sufficient to catalyze the condensation,^58^ and the resultant imines were neutralized before the characterization of their photoswitching properties. The isolated imines remained stable in the solid state for over two months and showed no signs of hydrolysis when dissolved in MeCN-d_3_ (*ca.* 2 mM) even after two weeks under ambient conditions. Alongside the AIPs, a reference compound, diphenylimine 1, was synthesized to underline the enhanced photoswitching properties of the AIPs, as summarized in Table 1.

**Table 1:** Summary of the photoswitching properties for AIPs and reference compound 1. The PSS values reported here were obtained from UV/vis measurements performed at 20 °C in MeCN. The values in parentheses under the PSS section do not refer to the PSS but the maximum measured % *Z* isomer; the PSS is not reached under these conditions due to the significant thermal back isomerization taking place during photoirradiation. Listed quantum yields (QYs) correspond to *E*-to-*Z* photoisomerization; *Z*-to-*E* values are in parentheses. Extended data on PSS and QYs at alternative wavelengths can be found in Table S4

	% *Z* at PSS				*t*_1/2_	QY (%)
	365 nm	405 nm	430 nm	470 nm	(20 °C)	365 nm
1^a^	−(1)^b^	—	—	—	0.5 s	—
2^a^	−(21)^b^	—	—	—	12.5 s	—
3	66	95	90	55	22.1 min	2 (2)
4	68	95	99	94	19.2 h	1 (1)

a Note that the short *t*_1/2_ values preclude the measurement of the % *Z* at the PSS under these conditions (Fig. S15).

b The maximum measurable % *Z* isomer recorded at 20 °C.

The X-ray crystal structure of *E*-4 revealed a non-planar conformation. A twist of 23° was noted in the smaller dihedral angle between the phenyl and imine (*Φ*_s_ (Ph)), while a 38° twist is observed for *Φ*_s_ (Pyr). This twist results from steric clashes between the imine bond and the *ortho*-substituents the adjacent rings. Notably, the steric clash between the *ortho*-pyrrolidine units and the imine distorts the imine from planarity by *ca.* 19° (Fig. 2d).

**Fig. 3:**
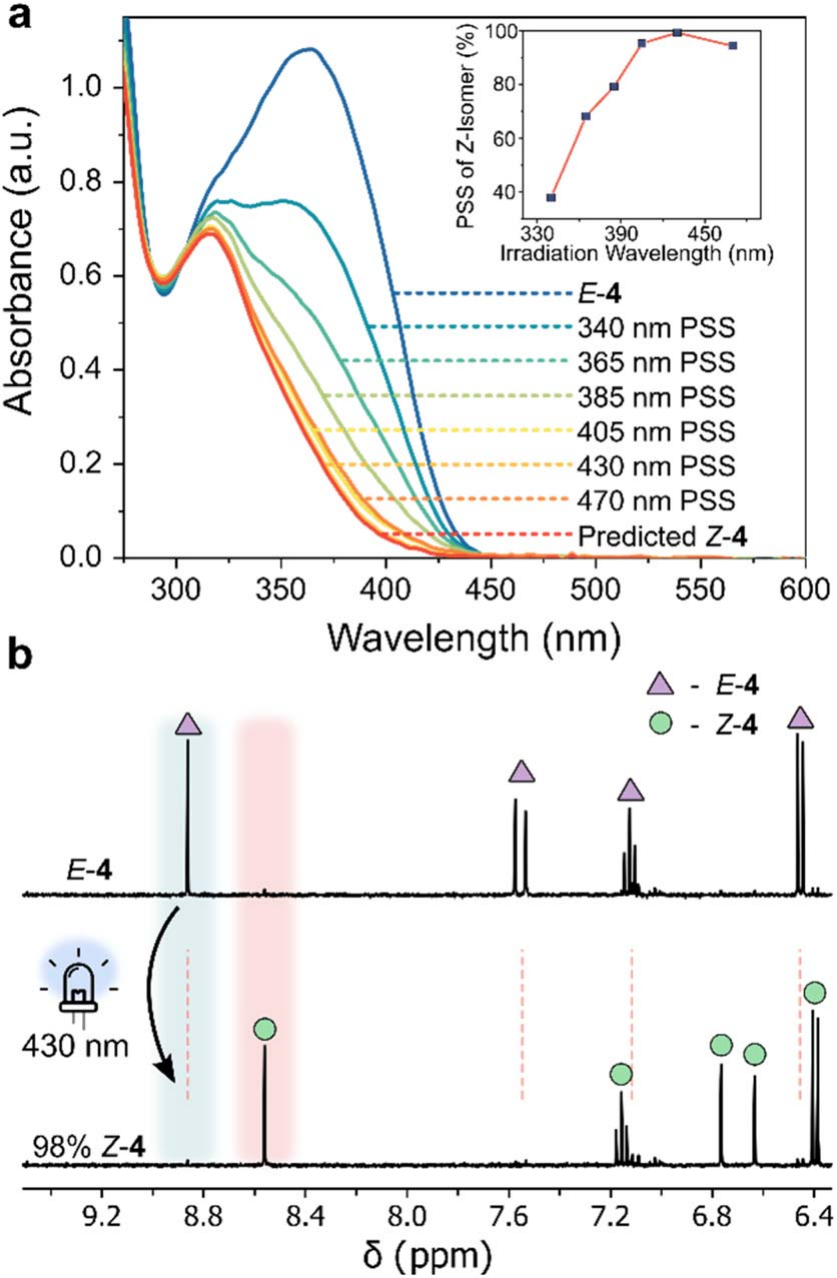
(a) UV/vis absorption spectra of 4 (170 μM, 20 °C, MeCN) at various photostationary states (PSS), and the spectrum of the *Z*-isomer obtained as described in Section 4.5 of the ESI.†^59^ (inset) *Z*-Isomer percentage generated at different irradiation wavelengths (Table S4†). (b) ^1^H NMR spectra (400 MHz, 298 K, CD_3_CN) of *E*-4 (triangle), highlighting the emergence of new signals attributed to *Z*-4 (circle) upon 430 nm photoirradiation.

Photoinduced *E*/*Z* isomerism was observed for the AIPs at room temperature using UV/vis spectroscopy, summarized in Table 1. The AIPs demonstrate T-type behavior, where the back reaction of the metastable state to the thermodynamically stable isomer occurs thermally. Notably, visible-light-induced negative photochromism was observed for 3 and 4 (Fig. 3a and S13†), whereby *λ*_max_(*E*) > *λ*_max_(*Z*). This specific type of photochromic behavior is advantageous in applications requiring large light penetration depths.^48^ The lowest energy absorption band in the UV/vis spectra is assigned to a π–π* transition, corroborated by TD-DFT studies (Section 5 of ESI†).

Examining the UV/vis absorption spectra revealed that *ortho*-aminated AIPs exhibit significant bathochromic shifts—up to 50 nm in the π–π* band—compared to the non-functionalized analogue 2 (Table S5†). Specifically, the pronounced 50 nm red-shift in the π–π* band of *E*-3 enabled *E*-to-*Z* photoisomerism with 470 nm light. Upon photoisomerization, we observed a hypsochromic shift in the π–π* transition, confirming negative photochromism. The largest separation between the π–π* bands of the two isomers occurred in 4, where both *ortho*-positions were aminated. This led to a 53 nm blue-shifted transition for the *Z*-isomer compared to its *E*-form. In contrast, compound 3, with a single *ortho*-aminated site, exhibited a smaller hypsochromic shift of 25 nm.

The AIPs demonstrated more complete *E*-to-*Z* photoswitching, evidenced by significantly larger compositions of the *Z*-isomer generated at the PSS compared to the maximum obtainable amount of the *Z*-isomer of 1 and the other previously reported arylimines (Table S3†). We quantified the *Z*-isomer composition at the PSS using UV/vis spectroscopy (Fig. 3a and S13†),^59^ and further corroborated these values with complementary NMR measurements (Fig. 3b and S14†). Near-complete *E*-to-*Z* switching (with ≥95% of *Z*-isomer at the PSS) was noted for 3 and 4 using visible light (Table 1, Fig. 3b, S13 and 14†). This is attributed to the bathochromically shifted π–π* transition, the large separation of this band between the *E* and *Z* isomers.^54^ Such quantitative *E*-to-*Z* photoswitching constitutes a significant achievement in the development of imine-based photoswitches.^54^ The measured quantum yields for both the forward and reverse photoswitching were found to be ≤10% (Section 4.2 and 4.7 of the ESI†).

To our surprise, the *Z*-AIPs displayed a markedly long thermal half-life, exemplified with 4 showing a *t*_1/2_ of 19.2 hours at 20 °C. This represents a four orders-of-magnitude increase in thermal stability compared to the most stable *Z*-arylimine reported so far and 10^5^ times longer than 1 (Table 1 and S3†). It is intriguing that *ortho*-amination of the phenyl ring with pyrrolidine led to an increase in the *t*_1/2_. This behavior contrasts that of the azo-based photoswitches, where *ortho*-amination typically decreases the thermal stability of the *Z*-state.^55,56^ This deviation from established trends highlights unique properties of the AIPs, justifying further research into the structure–function relationships of these imine-based photoswitches.

**Fig. 4:**
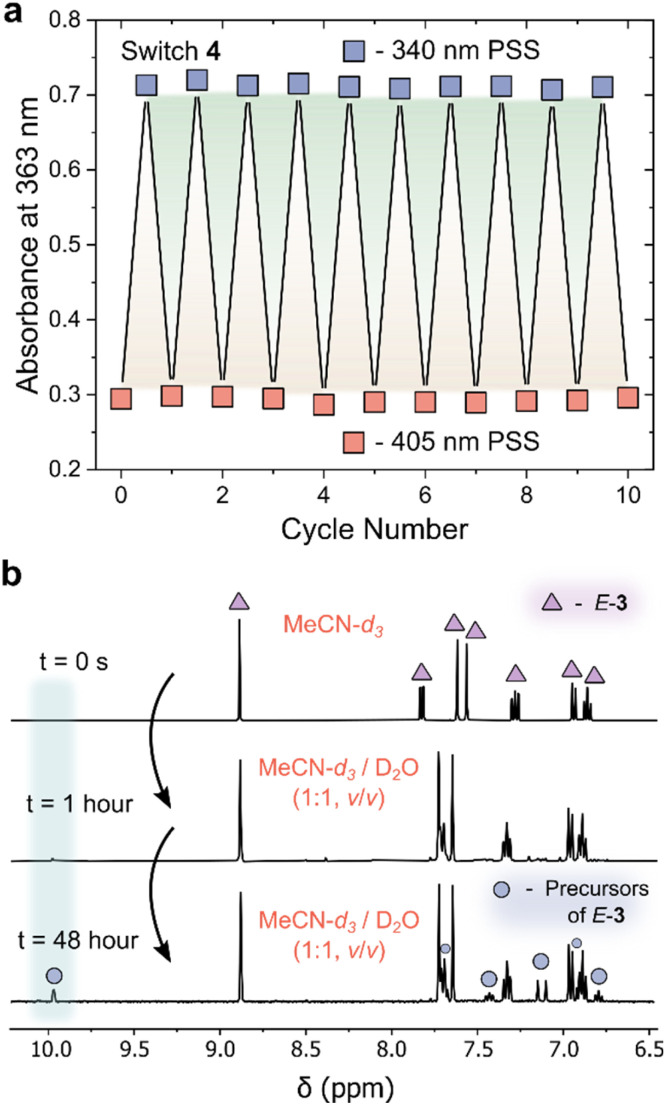
(a) Plot showing the fatigue resistance of AIP 4 to repeated photoisomerism with 340 nm and 405 nm irradiation. No signs of degradation are observed over 10 switching cycles. (b) ^1^H NMR spectra (400 MHz, 298 K) of *E*-3 (triangle), highlighting the emergence of new signals attributed to amine and aldehyde precursors of *E*-3 (square) over time in a 1 : 1 (v/v) solution of MeCN-d_3_ and D_2_O with an initial concentration of 3 of 2 mM. The shaded blue region at 10 ppm indicated the emergence of the aldehyde signal.

In addition to an enhanced thermal stability of the *Z*-isomer, AIPs 3 and 4 displayed excellent resistance to fatigue: no signs of degradation were observed in the UV/vis absorption spectra after 10 photoswitching cycles (Fig. 4a and S16†). Another important consideration is the hydrolytic stability of the imines towards water. The NMR spectra of AIP 3 (2 mM) was recorded in a MeCN-d_3_/D_2_O mixture after 1 hour and 48 hours (Fig. 4b). Even in this large excess of D_2_O, the imine remained surprisingly stable, showing only 16% of the imine hydrolyzing back to the aldehyde and amine precursors after 48 hours at 25 °C. The stability of these imines to the presence of water is attributed to the improved nucleophilicity of the aminopyrazole unit.

The influence of different solvents on the photoswitching behaviour of AIP 4 was examined (Section 4.8 of ESI†). UV/vis absorption spectra and the proportion of *Z*-isomer at the PSS showed minimal variation across the solvents studied (which included toluene, 1,4-dioxane, tetrahydrofuran, dichloromethane, dimethyl sulfoxide, and acetonitrile, see Table S8 and Fig. S23†). For instance, the maximum absorption wavelength of the *E*-isomer's lowest energy band shifted by less than 10 nm among the solvents tested. However, the choice of solvent markedly influenced the thermal half-life of the *Z*-to-*E* isomerization (Fig. S24 and Table S8†). Dichloromethane resulted in the shortest *t*_1/2_ (3.2 h), while acetonitrile showed the longest (19.2 h), indicating a 4 kJ mol^−1^ difference in the apparent thermal barrier to reversion. Generally, we observed that aprotic solvents with higher empirical solvent parameter, *E*^N^_T_ values,^60^ correlated with a longer *t*_1/2_, suggesting that the *Z*-to-*E* isomerization likely proceeds through an inversion transition state.^61^

**Fig. 5:**
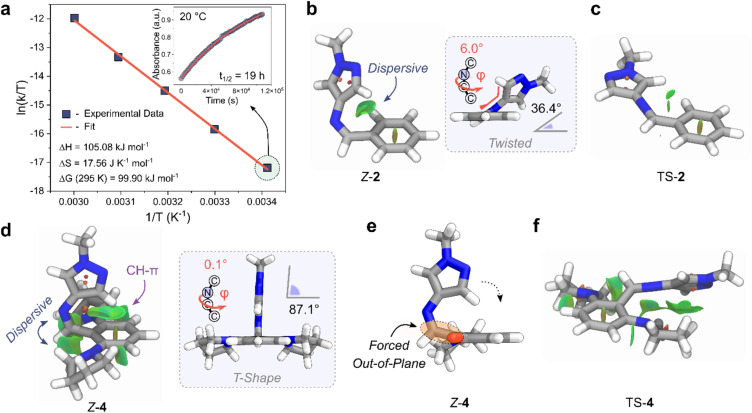
(a) Eyring plot of the longest-lived *Z*-AIP, 4. (inset) Rate of thermal reversion of the *Z*-to-*E* isomer at 20 °C. (b) Geometry-optimized *Z*-2 highlighting green NCI surfaces between the pyrazole and phenyl rings. (inset) Display of the twisted geometry. (c) Geometry-optimized TS-2 with NCI surfaces displayed. (d) Geometry-optimized *Z*-4 showing large NCI interactions between the imine and the *ortho*-pyrrolidine units, as well as CH–π interactions between the CH of the pyrazole and the phenyl ring. (inset) View showing the T-Shape conformation. (e) Alternate view of *Z*-4; the pyrrolidine projecting out of the page is depicted as a red stub for clarity. (f) Geometry-optimized structure of TS-4 with NCI surfaces displayed.

We investigated the mechanism behind the significant stabilization of the *Z*-isomer using DFT, adopting the ωB97X-D4 functional in combination with a def2-TZVPP basis set, and a CPCM solvation model for acetonitrile (Section 5 of the ESI†).^62,63^ Ground-state optimized structures revealed a twisted conformation for *Z*-2 and *Z*-3, while *Z*-4 adopted a T-shaped structure (Fig. 5b and d). Significant stabilizing non-covalent interactions (NCIs) were observed for the *Z*-isomer, illustrated graphically by the NCI surfaces in Fig. 5.^64^ The impact of dispersive interactions was quantified by evaluating the difference in the dispersive energy between the *Z* and *E* isomers (referred to as *E*^D4^_*Z*–*E*_) with Grimmes DFT-D4 approach.^62^

The NCI interactions are particularly pronounced for *ortho*-aminated derivatives with *E*^D4^_*Z*–*E*_ values as high as +17.45 kJ mol^−1^ for 3 compared to +6.97 kJ mol^−1^ for 2. In the case of 4, the additional steric bulk of the two flanking pyrrolidine units forces the structure to adopt a T-shape conformation by minimizing the steric repulsion between the imine hydrogen and the pyrrolidine units (Fig. 5d and e).^54^ Closer inspection of the structure revealed that the carbon atom of the imine bond in *Z*-4 is also forced out of plane relative to the phenyl ring due to the steric requirements of the *ortho*-pyrrolidine units, similar to the crystal structure of *E*-4 (Fig. 2d and 5e). Importantly, this distortion brings the CH of the pyrazole closer to the centroid of the phenyl ring (distance of 2.3 Å). The resulting conformation enhances the NCI stabilization, favoring the formation CH–π interactions between the pyrazole and the phenyl ring, while maximizing the dispersive interactions with the flanking pyrrolidine motifs.

For the *Z*-to-*E* thermal reversion process, the AIPs follow an inversion trajectory with linearization of the C–N=C bond (Fig. S26†).^65^ Stabilizing NCIs are also observed in the TS structures, and are more pronounced for the *ortho*-functionalized derivatives (Fig. 5c and f and Section 5 ESI†). Given this, we infer that the increase in *t*_1/2_ predominantly originates from an increased stabilization of the *Z*-isomer due to NCI interactions as opposed to a destabilization of the TS. Specifically, the near 90° arrangement (T-Shape) of the pyrazole ring with the phenyl ring of *Z*-4 is positioned to conformations of 2 and 3. Additionally, unlike azo-based photoswitches, the absence of adjacent non-bonding lone pairs in the imine bond prevents any additional destabilizing electron repulsion in the HOMO when incorporating the electron-donating pyrrolidine motifs.

Taken together, the key observations reported here for this new class of dynamic-covalent photoswitch include: (i) the π–π* absorption transition can be red-shifted by *ortho*-aminating the phenyl ring. This bathochromic shift allows for visible light operation, widening the scope of practical applications. (ii) Maximizing NCIs through *ortho*-functionalization of the phenyl ring markedly stabilizes the *Z*-isomer, resulting in enhanced *t*_1/2_. This contrasts with azo-based systems, where *ortho*-amination typically reduces *Z*-isomer stability. (iii) Unlike azo-based switches, the absence of lone pair repulsion in the C=N bond of the *Z*-AIPs facilitates the inclusion of electron donating units without significantly destabilizing the HOMO.

Setting this work in context with other photoswitches that exhibit *E*/*Z* isomerism, the AIPs display a distinct combination of properties. While the geometric change from the relatively planar *E*-isomer to the twisted or T-shaped *Z*-isomer in AIPs resembles azo-based photoswitches, the effects of incorporating a pyrazole ring and *ortho*-amination are different. Notably, in contrast to arylazopyrazoles,^46,54^ which show markedly extended half-lives, near-quantitative PSSs, and positive photochromism compared to azobenzene, AIPs do not exhibit a significant improvement by the sole incorporation of the pyrazole ring, as highlighted for 2. However, the introduction of *ortho*-pyrrolidine units, typically reducing the *t*_1/2_ in azo-based switches,^55,56^ stabilizes the metastable state of the AIPs for the reasons presented above, and imparts negative photochromism to the switch. These properties, paired with the ease of synthesis, accessibility of precursors, and reversibility of the imine bond, set the AIPs apart from the azo-based photoswitches.

The arylhydrazones also contain a dynamic-covalent motif (C=N–NH),^66^ albeit less dynamic than an imine bond, and can undergo *E*/*Z* photoisomerism. Unlike the AIPs however, the photochromic motif of the arylhydrazones is comprised of a six-membered H-bonded ring system with an acidic NH site,^66^ thus different strategies are used for tuning the stability of their metastable state. Regarding the geometric change associated with *E*/*Z* isomerism, the arylhydrazones are more planar compared to the larger three-dimensional change observed for the AIPs.^66^ Thus, AIPs are arguably more suited for applications requiring a greater dynamicity or a larger geometric change upon photoisomerism, such use in self-assembled photoresponsive organic capsules.^67,68^ Other recently reported classes of photoswitch that operate around a C=N bond include the iminothioindoxyl^35^ and phenylimino indolinone^3^ photoswitches, though these systems have not exhibited dynamic-covalent chemistry. These imino-based switches display negative photochromism and a *t*_1/2_ on the μs to ms time scale.^3,35^

Collectively, the AIPs stand out as dynamic-covalent photoswitches that exhibit large geometric changes upon isomerization.^69^ Moreover, the simplicity and availability of their precursors, paired with quantitative imine-formation and useful photoswitching properties makes them enticing photoswitches in their own right.

## Conclusions

In conclusion, we have successfully addressed the key challenges that previously hindered arylimines from being utilized as effective photoswitchable molecules. Notably, we have identified structural motifs that exhibit: (i) negative photochromism; (ii) photoisomerization with visible light (470 nm); (iii) display near-quantitative *E*-to-*Z* photoisomerism (>95% *Z*-isomer); (iv) excellent fatigue resistance (*v*) and prolonged thermal half-lives up to 19.2 hours at room temperature. Key to these improved properties is the use of an aminopyrazole subcomponent, combined with *ortho*-amination of the phenyl ring, which synergistically extended the thermal stability of the metastable state and red-shift the UV/vis absorption. The increased *t*_1/2_ is attributed to the strong NCI stabilization in the *Z*-isomer. The negative photochromism distinguishes AIPs from azo-analogues as the former exhibits no competing *Z*-to-*E* photoisomerism at the longest wavelengths of electronic absorption. Moreover, the negative photochromic behavior of these switches, in particular, presents advantages in applications requiring large light penetration depths,^48,53^ such as molecular solar-thermal fuels (MOST).^48^ It is also important to consider the scalability and accessibility of the material, and its precursors, when evaluating suitability for an application. Thus, the quantitative assembly and availability of precursors render AIPs as attractive candidates. To this end, our laboratory is currently investigating new imine-based photoswitches and working towards the utilization of these photochromic linkages in larger organic architectures and adaptive dynamic-covalent networks, these results will be reported in due course.

## Data availability

The experimental procedures, analytical data and computational details are available within the manuscript and its ESI.† CCDC number 2293811 contains the supplementary crystallographic data for AIP *E*-4. Data are available upon request from the authors.

## Author contributions

J. W. synthesized the subcomponents and imines, characterized the imines by NMR, MS, and UV/vis, and performed the photoswitching experiments. S. T. assisted in the synthesis of the subcomponents and imines, and their molecular characterization. L. K. conducted the computational investigations. O. N. collected and solved the crystallographic data. J. L. G. conceived the project, supervised the research, and wrote the manuscript, with input from all the authors.

## Conflicts of interest

There are no conflicts to declare.

## Supplementary Material

SC-015-D3SC05841G-s001

SC-015-D3SC05841G-s002
