# Supplementary material for: Photoswitchable imines: aryliminopyrazoles quantitatively convert to long-lived Z-isomers with visible light
Source: Chem Sci. 2024 Feb 14;15(11):3872–8. doi: 10.1039/d3sc05841g (PMC10935669; doi:10.1039/d3sc05841g)
Supplement: SC-015-D3SC05841G-s001 [file SC-015-D3SC05841G-s001.pdf]

Electronic Supplementary Material (ESI) for Chemical Science.
This journal is © The Royal Society of Chemistry 2024

Supporting Information:

Photoswitchable Imines: Aryliminopyrazoles Quantitatively Convert to Long-Lived *Z*-Isomers with Visible Light

Jiarong Wu,[a,b] Lasse Kreimendahl,[a] Suyuan Tao,[a,b] Olga Anhalt[b] and

Jake L. Greenfield[a,b]*

[a] *Institut für Organische Chemie, Universität Würzburg, 97074 Würzburg, Germany*

[b] *Center for Nanosystems Chemistry (CNC), Universität Würzburg, 97074 Würzburg, Germany*

* Corresponding Author (Jake.Greenfield@uni-wuerzburg.de)

Table of Contents

1. Materials and Methods 3
   1.2 UV/vis and Photoswitching Studies 4
      1.2.1 Diode Array Setup 4
2. X-ray Crystallography 6
3. Synthesis and Characterisation 7
4. Supporting and Characterisation Data 14
   4.1 Figure of Merit Table 14
   4.2 Overview of Photoswitching Properties 15
   4.3 Extinction Coefficient 16
   4.4 Thermodynamic Properties 18
      4.4.1 Thermal Half-Life Data 19
      4.4.2 Fitting of Rate Constants to the Eyring Equation 23
   4.5 Photostationary States (PSS) 24
   4.6 Fatigue Resistance 27
   4.7 Quantum Yields 28
   4.8 Influence of Solvent 35
5. Computational Studies 38
   5.1 Conformer Search and Geometry Optimisation of *E* and *Z* isomers 38
   5.2 Conformer Search and Geometry Optimisation of Transition States 39
   5.3 Theoretical Investigation of Thermal Isomerisation Rates and Half-Lives 41
   5.4 Non-Covalent Interaction (NCI) Analysis 42
   5.5 Excited State Analysis 42
   5.6 Theoretical Data for Photoswitch **1** 44
   5.7 Theoretical Data for Photoswitch **2** 45
   5.8 Theoretical Data for Photoswitch **3** 46
   5.9 Theoretical Data for Photoswitch **4** 47

References 48

1. Materials and Methods

All reagents and solvents were purchased from commercial suppliers and used without further purification, unless specified. Solvents were dried with a commercial solvent purification system PS-M6-6/7-En from Inert Technologies. All synthesised photoswitches were stored under N_2, kept dark and dried under high-vacuum for at least 24 h prior to use.

UV/Vis Absorption. Spectra were recorded on a Jasco V-770 spectrophotometer, equipped with a Peltier cooling system (PAC-743R), or a diode array setup as detailed below in Section 1.2.1. Standard 10 mm pathlength quartz glass cuvettes (Fluorescence Cuvettes) from Hellma were used and a stirring bead was added. Measurements were run at 293.15 K, unless otherwise stated. Solutions for the UV/vis measurements were made using spectroscopy grade dry degassed solvents. A background measurement containing only the solvent used (or blank substrate for solid-state measurements) was recorded before measuring samples. This background was subtracted from the sample data using Origin Software.

High-Resolution Mass Spectrometry (HR-MS). Electrospray ionisation (ESI) HR-MS spectrograms were recorded on a Bruker Daltonics microTOF focus instrument with a resolution of 18.000 FWHM. Samples were prepared in MeCN with 1% formic acid and the instrument was run in positive mode.

NMR Spectroscopy. NMR spectra were recorded at 298 K using a Bruker Avance HD III 400 MHz spectrometer automatically tuned and matched to the correct operating frequencies. The ^{13}C NMR spectra are broad-band proton decoupled. TopSpin 4.2 and MestReNova 14.3.2 were used to apply phase and baseline corrections. ^{1}H and ^{13}C NMR spectra were referenced to the residual solvent peak, and the ^{19}F NMR spectra of organic molecules were referenced to hexafluorobenzene at –164.9 ppm. Signals are reported in terms of chemical shift (ppm) and coupling constants (Hz). Abbreviations for multiplicity are as follows: s, singlet; d, doublet; t, triplet; m, multiplet; br, broad; hept, heptet.

Quantum Yield of Photoswitching. Ferrioxalate actinometry was used to determine the photon flux from the 340, 365, 385, 405, 430 nm LEDs detailed in Section 4.7 and the photon flux from the 470 nm LED was determined using Aberchrome 670 following a previously reported procedure.[6] The $\square_{ZE}$ and $\square_{EZ}$ photoisomerization for the aryl imine photoswitches were determined using previously reported literature procedure and the calculation was performed using their provided software.[6] Collimated light was used when determining the photon flux, $\square_{ZE}$, and $\square_{EZ}$.

1.2 UV/vis and Photoswitching Studies

The UV/vis absorption spectrum of the photoswitches reported in this paper were collected using either a JASCO V-770 spectrophotometer with a PAC-743R Peltier temperature controller or a home-built diode array setup (schematically shown below in Figure S1).

1.2.1 Diode Array Setup

The diode array setup schematically shown in Figure S1 was employed for Photostationary State (PSS), thermal half-life and quantum yield measurements. The setup consists of an Ocean Insight DH-2000-FHS-DUV-TTL light source (190-2500 nm), connected to a Quantum Northwest QPod sample holder by 25 cm long premium fibre optics (Ocean Insight QP400-025-SR-BX). The temperature of the QPod sample holder was controlled using a Quantum Northwest TC 125 temperature controller, which also controlled the stirring. An Ocean Insight Flame-S-XR1-ES diode array spectrometer was used to measure the absorbance spectrum of the sample. Pin-hole slits were employed as shown in the schematic along with an optical filter at the light source to reduce the probe light intensity (Thermo Oriel 50550).

LEDs of various wavelengths (see Table S1 below) were employed to induce photoisomerization. These LEDs were fitted with adjustable collimation adapters supplied by ThorLabs (either a SM1U with an LA4052-UV Fused Silica Plano Convex Lens with an anti-reflective coating 245-400 nm or a SM1U25-A with an anti-reflective coating 350-700 nm, depending on the wavelength of the LED used). The LEDs were operated at a constant current mode controlled by a ThorLabs DC2200 LED driver.

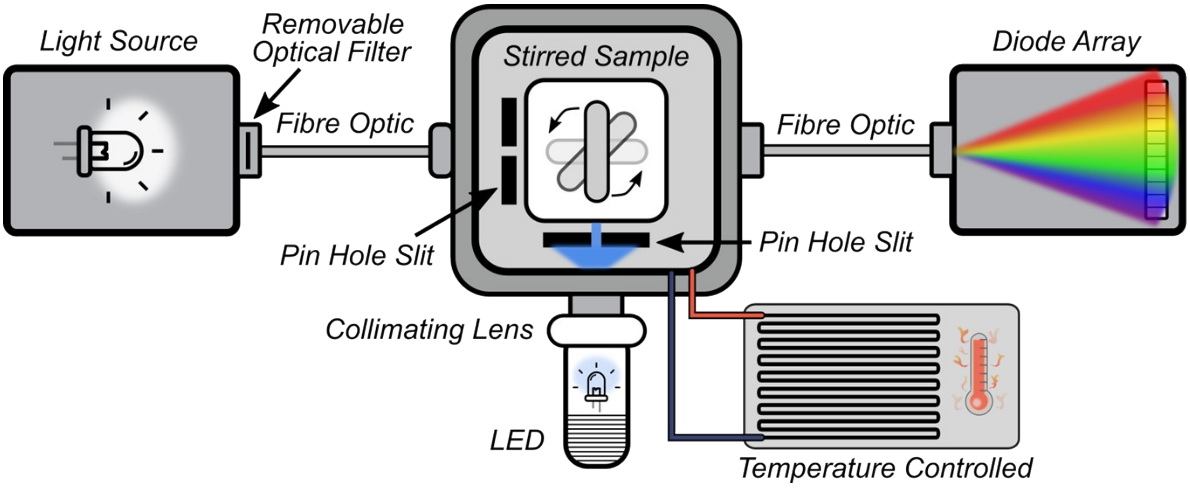

Figure S1. Schematic representation of the diode array setup up to measure the UV/vis absorption spectra and to perform photoswitching studies.

Table S1. Table displaying the models of ThorLabs mounted LEDs used in this study along with the nominal emission wavelength, the wavelength in which emission appears brightest to the human eye, and the bandwidth (full width at half maximum, FWHM).

LED model number (ThorLabs)	Wavelength (nm)	Bandwidth/FWHM (nm)
M340L5	340	10
M365L3	365	9
M385L3	385	11
M405L4	405	13
M430L5	430	17
M470L5	470	28
M505L4	505	37
M590L4	590	15

2. X-ray Crystallography

The collection of crystallographic data of the aryl imine photoswitches was obtained by using a Bruker D8 Quest diffractometer with a Photon II CMOS detector and Cu Kα radiation (λ = 1.54178 Å). A solution of the structure was performed with direct methods, refinement with the SHELX software package and expanding using Fourier techniques.[1] Nonhydrogen atoms were refined anisotropically while hydrogen atoms were positioned onto idealized positions and included in calculations of structure factors. CCDC 2293811 contains the supplementary crystallographic data for this paper. These data can be obtained free of charge from The Cambridge Crystallographic Data Centre via www.ccdc.cam.ac.uk/data_request/cif.

Table S2. X-ray structure characteristics for AIP photoswitch **4**.

		E-**4**
	Empirical Formula	$C_{19}H_{25}N_5$
	$M_{empirical}$ (g mol^{-1})	323.44
	Wavelength (Å)	1.54178
	T (K)	100
Crystal Description	Color	Yellow
	Habit	Block
	Crystal System	Monoclinic
	Space Group	C 2/c
Unit Cell Dimensions	a (Å)	31.026(4)
	b (Å)	6.7327(4)
	c (Å)	19.7204(19)
	α (°)	90
	β (°)	124.143(7)
	γ (°)	90
	Volume (Å^3)	3409.3(6)
	Z	8
	$\rho_{alc.}$ (g cm^{-3})	1.260
	F(000)	1392
	Range of θ (°)	3.442 72.172
	Goodness of Fit	1.047
	CCDC	2293811

3. Synthesis and Characterisation

The imine condensation reactions proceed quantitatively however the 1-methyl-1H-pyrazol-4-amine is prone to degradation and the quality of material provided by different suppliers varies. To alleviate this issue and simplify the synthesis, an excess of the amine was used in the condensation reactions.

<u>Synthesis of 2-(pyrrolidin-1-yl)benzaldehyde, S1</u>

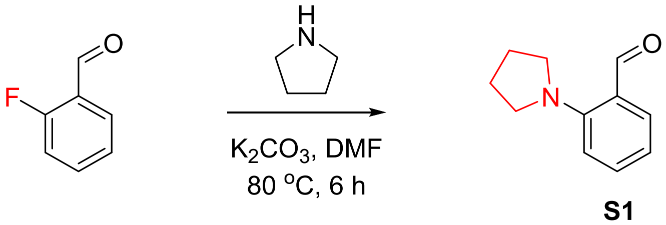

S1

To an oven dried flask was added 2-fluorobenzaldehyde (2.00 g, 16.1 mmol, 1 eq), K_2CO_3 (4.45 g, 32.2 mmol, 2 eq) and 10 mL of anhydrous DMF. To this suspension was added pyrrolidine (1.15 g, 16.1 mmol, 1 eq) and the mixture was then left to stir at 80 °C for 6 h under an N_2 atmosphere. The mixture was then filtered under gravity and the filtrate was concentrated in *vacuo*. The resulting oil was purified by column chromatography on silica gel using an eluent of 9:1 cyclohexane:ethyl acetate. The product **S1** was dried under high vacuum affording a yellow oil (2.60 g, 92% yield). ^{1}H NMR (400 Hz, 298 K, Chloroform-*d*) δ 9.98 (s, 1H), 7.59 (dd, *J* = 7.8, 1.8 Hz, 1H), 7.26 (ddd, *J* = 8.7, 7.0, 1.8 Hz, 1H), 6.73 – 6.66 (m, 2H), 3.26 – 3.22 (m, 4H), 1.89 – 1.84 (m, 4H); ^{13}C NMR (101 MHz, 298 K, Chloroform-*d*): δ 189.97, 149.89, 134.12, 132.93, 122.94, 116.36, 114.49, 52.36, 25.88 ppm; HR ESI-MS (MeCN) for $[C_{11}H_{13}ON+H]^+$: m/z calcd: 176.1070; found: 176.1071; 0.6 ppm error.

Synthesis of 2,6-di(pyrrolidin-1-yl)benzaldehyde, S2

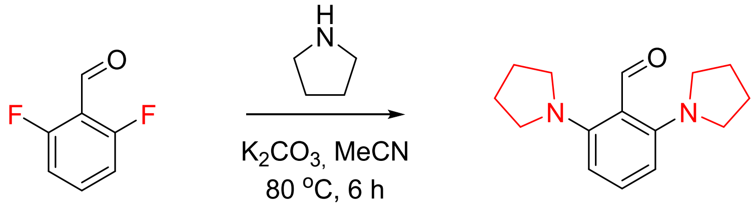

S2

To an oven dried flask was added 2,6-difluorobenzaldehyde (1.00 g, 7.0 mmol, 1 eq), K_2CO_3 (1.94 g, 14.1 mmol, 2 eq) and 30 mL of anhydrous MeCN. To this suspension was added pyrrolidine (2.00 g, 28.1 mmol, 4 eq) and the mixture was then left to stir at 80 °C for 6 h under an N_2 atmosphere. The mixture was then filtered under gravity and the filtrate was concentrated in *vacuo*. The resulting oil was purified by column chromatography on silica gel using an eluent of 9:1 cyclohexane:ethyl acetate. The product **S2** was dried under high vacuum affording an orange solid (1.44 g, 84% yield). ^{1}H NMR (400 Hz, 298 K, Chloroform-*d*) δ 9.74 (s, 1H), 7.18 – 7.09 (m, 1H), 6.13 (d, J = 8.2 Hz, 2H), 3.49 – 3.41 (m, 8H), 1.99 – 1.91 (m, 8H); ^{13}C NMR (101 MHz, 298 K, Chloroform-*d*): δ 184.71, 154.82, 135.14, 129.31, 111.20, 102.22, 77.36, 52.68, 51.76, 51.65, 25.94, 25.80, 25.43 ppm; HR ESI-MS (MeCN) for $[C_{15}H_{20}N_2O+H]^+$: m/z calcd: 245.1648; found: 245.1644; 1.6 ppm error.

Synthesis of *(E)-N,*1-diphenylmethanimine, 1

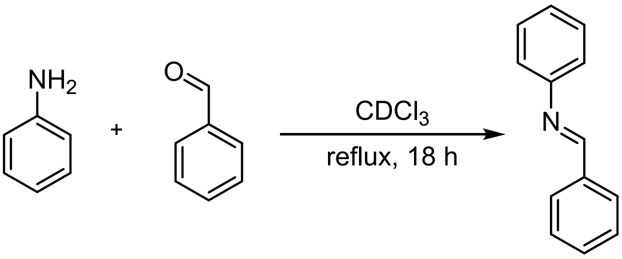

1

In oven-dried glassware charged with 3 Å molecular sieves, aniline (114.1 mg, 1.2 mmol, 1.5 eq) and benzaldehyde (100.0 mg, 0.9 mmol, 1.0 eq) were dissolved in $CDCl_3$ (2.0 mL) under N_2. The mixture was heated to reflux for 18 hours under N_2, and reaction completion was determined by TLC. The solvent was then removed in vacuo. The solid was purified via flash-chromatography using EtOAc with 1% Triethylamine to afford product **1** as white solid (quantitative reaction by ^1H NMR in $CDCl_3$, isolated 135.9 mg after purification, isolated yield of 83%). ^1H NMR (400 MHz, 298 K, MeCN-d3) δ 8.55 (s, 1H), 7.95 – 7.90 (m, 2H), 7.56 – 7.48 (m, 3H), 7.45 – 7.39 (m, 2H), 7.28 – 7.22 (m, 3H) ppm; ^{13}C NMR (101 MHz, 298 K, MeCN-d3): δ 161.56, 153.00, 137.44, 132.35, 130.20, 129.81, 129.58, 126.91, 121.80 ppm; HR ESI-MS (MeCN) for $[C_{13}H_{11}N+H]^+$: m/z calcd: 182.0964; found: 182.0968; 2.2 ppm error.

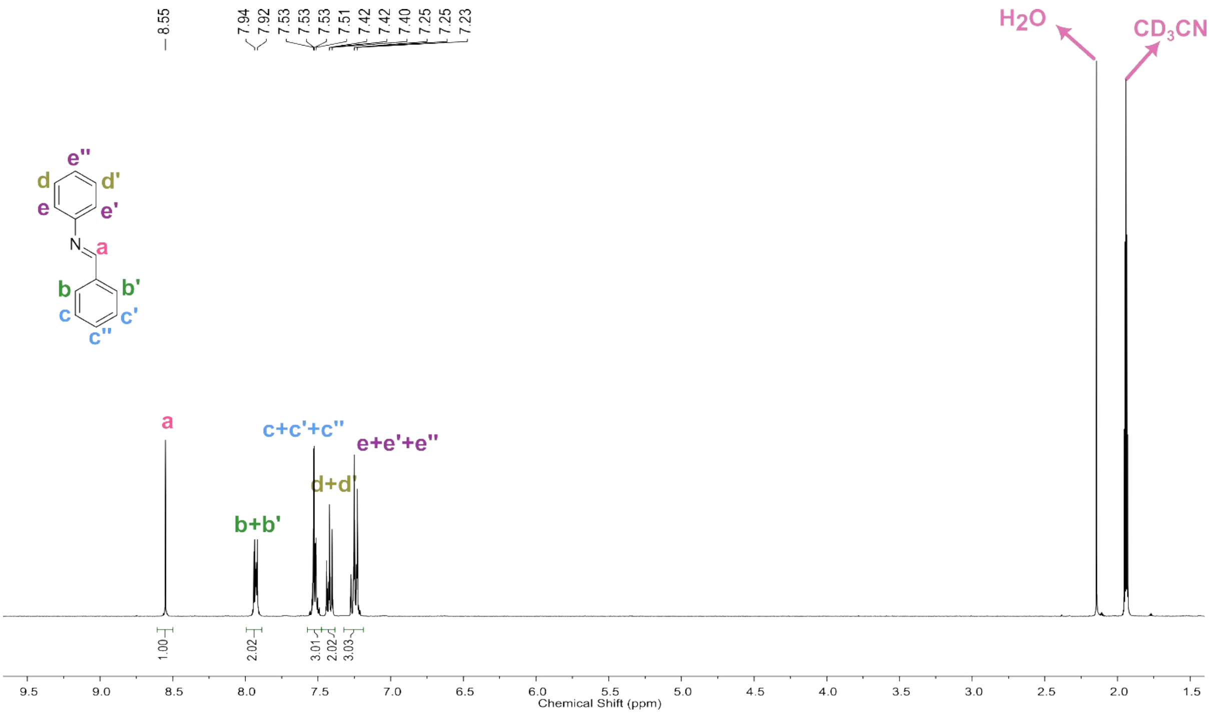

Figure S2. ^1H NMR (400 Hz, 298 K, MeCN-d3) of photoswitch **1**.

Synthesis of (*E*)-*N*-(1-methyl-1*H*-pyrazol-4-yl)-1-phenylmethanimine, 2

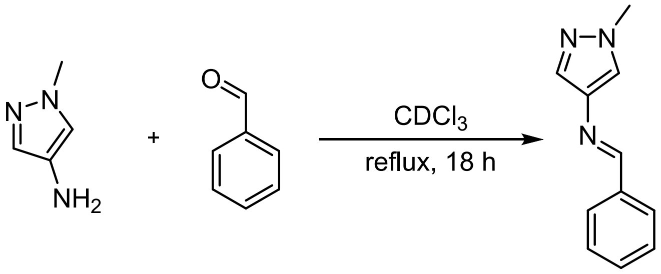

2

In oven-dried glassware charged with 3 Å molecular sieves, 4-amino-1-methylpyrazole (137.3 mg, 1.4 mmol, 1.5 eq) and benzaldehyde (100.0 mg, 0.9 mmol, 1.0 eq) were dissolved in $CDCl_3$ (2.0 mL) under N_2. The mixture was heated to reflux for 18 hours under N_2, and reaction completion was determined by TLC. The solvent was then removed in vacuo. The solid was purified via flash-chromatography using EtOAc with 1% Triethylamine to afford product **2a** as yellow crystalline solid (quantitative reaction by ^1H NMR in $CDCl_3$, isolated 158.4 mg after purification, isolated yield of 95%). ^1H NMR (400 Hz, 298 K, MeCN-d3) δ 8.64 (s, 1H), 7.85-7.81 (m, 2H), 7.66 (d, *J* = 0.8 Hz, 1H), 7.62 (d, *J* = 0.8 Hz, 1H), 7.48-7.45 (m, 3H), 3.84 (s, 3H) ppm; ^{13}C NMR (101 MHz, 298 K, MeCN-d3): δ 158.07, 137.90, 136.78, 132.08, 131.59, 129.72, 128.79, 125.29, 39.65 ppm; HR ESI-MS (MeCN) for $[C_{11}H_{11}N_3+H]^+$: m/z calcd: 186.1026; found 186.1026; 0.2 ppm error.

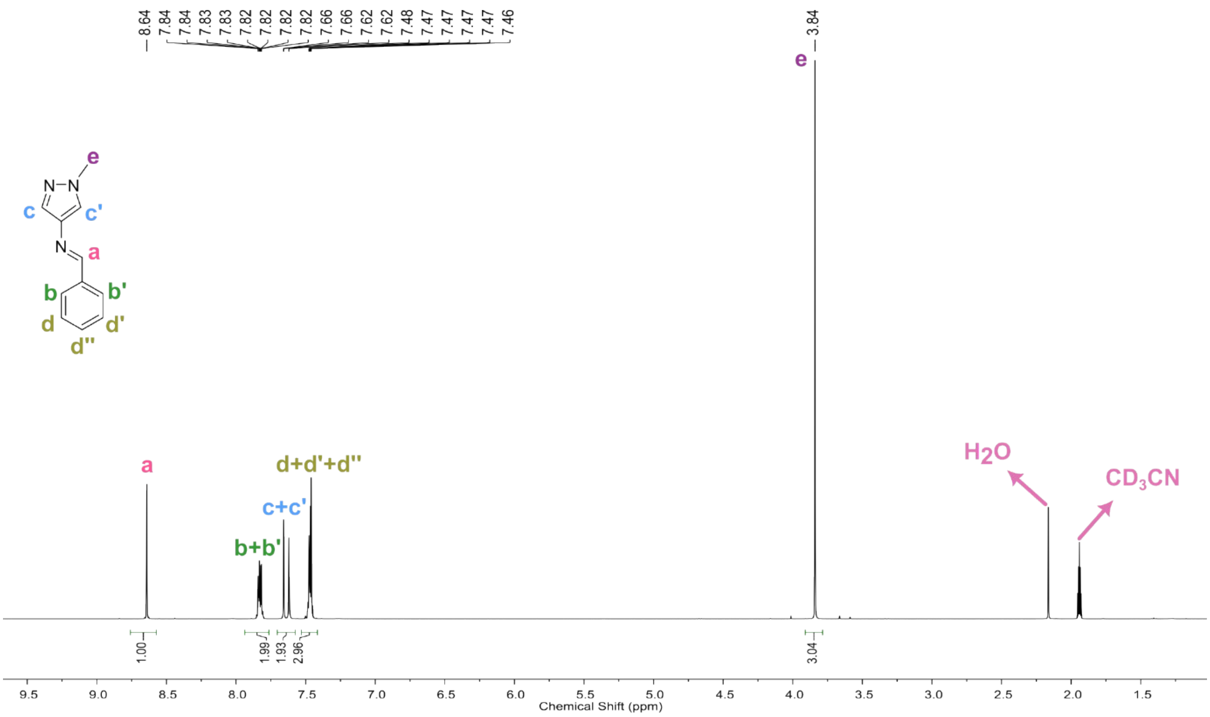

Figure S3. ^1H NMR (400 Hz, 298 K, MeCN-d3) of AIP **2**.

Synthesis of (*E*)-*N*-(1-methyl-1*H*-pyrazol-4-yl)-1-(2-(pyrrolidin-1-yl)phenyl)methanimine, 3

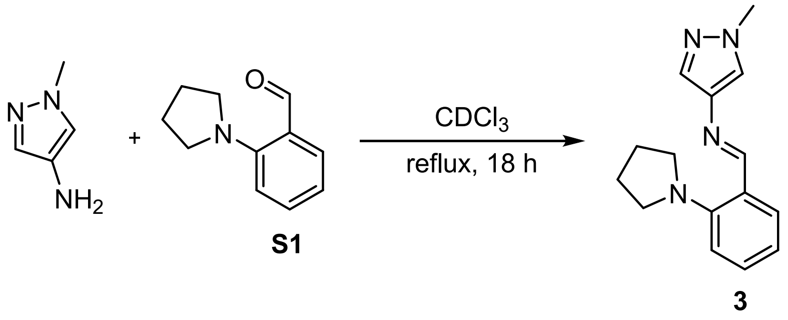

In oven-dried glassware charged with 3 Å molecular sieves, 4-amino-1-methylpyrazole (83.1 mg, 0.9 mmol, 1.5 eq) and **S1** (100.0 mg, 0.6 mmol, 1.0 eq) were dissolved in $CDCl_3$ (2.0 mL) under N_2. The mixture was heated to reflux for 18 hours under N_2, and reaction completion was determined by TLC. The solvent was then removed in vacuo. The solid was purified via flash-chromatography using EtOAc with 1% Triethylamine to afford product **3** as yellow oil (quantitative reaction by ^{1}H NMR in $CDCl_3$ isolated 141.4 mg after purification, isolated yield of 93%). ^{1}H NMR (400 Hz, 298 K, MeCN-d3) δ 8.89 (s, 1H), 7.83 (dd, J = 7.8 Hz, 1H), 7.59 (d, J = 21.5 Hz, 2H), 7.38 – 7.22 (m, 1H), 6.94 (d, J = 9.3 Hz, 1H), 6.86 (t, J = 7.4 Hz, 1H), 3.83 (s, 1H), 3.29 (m, 4H); ^{13}C NMR (101 MHz, 298 K, MeCN-d3): δ 157.71, 151.48, 137.80, 131.96, 131.83, 129.11, 126.34, 124.64, 119.92, 116.44, 53.81, 45.80, 39.59 ppm; HR ESI-MS (MeCN) for $[C_{15}H_{18}N_4+H]^+$: m/z calcd: 255.1604; found 255.1605; 0.3 ppm error.

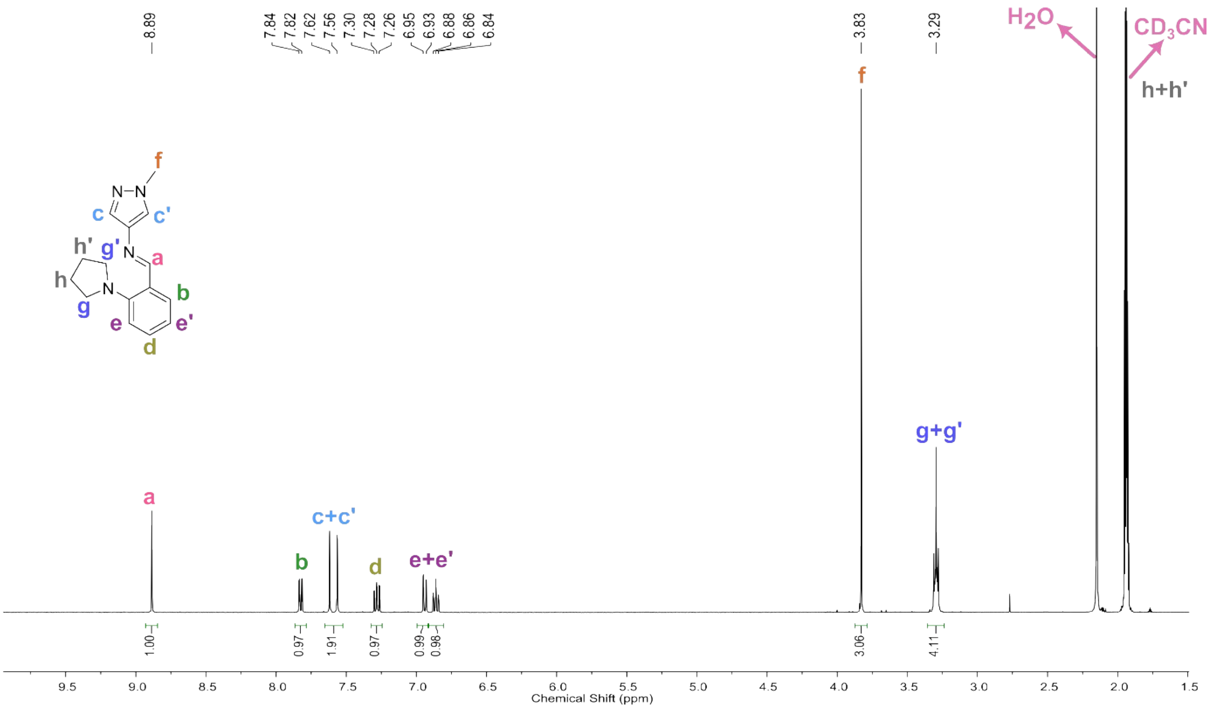

Figure S4. ^{1}H NMR (400 Hz, 298 K, MeCN-d3) of AIP **3**.

Synthesis of (*E*)-1-(2,6-di(pyrrolidin-1-yl)phenyl)-N-(1-methyl-1H-pyrazol-4-yl)methanimine, 4

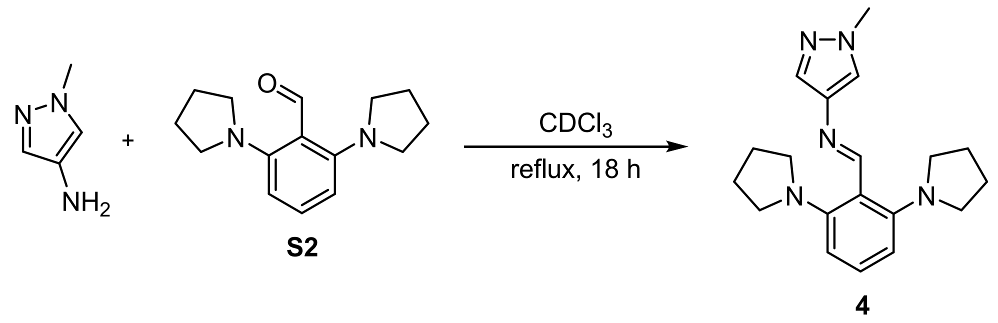

In oven-dried glassware charged with 3 Å molecular sieves, 4-amino-1-methylpyrazole (59.7 mg, 0.6 mmol, 1.5 eq) and **S2** (100.0 mg, 0.4 mmol, 1.0 eq) were dissolved in $CDCl_3$ (2.0 mL) under N_2. The mixture was heated to reflux for 18 hours under N_2, and reaction completion was determined by TLC. The solvent was then removed in vacuo. The solid was purified via flash-chromatography using EtOAc with 1% Triethylamine to afford product **4** as yellow solid (quantitative reaction by ^1H NMR in $CDCl_3$, isolated 105.9 mg after purification, isolated yield of 82%). ^1H NMR (400 Hz, 298 K, MeCN-d3) δ 8.83 (s, 1H), 7.55 – 7.50 (dd, 2H), 7.09 (t, 1H), 6.42 (d, 2H), 3.82 (s, 3H), 3.18 – 3.14 (m, 8H), 1.88 – 1.85 ppm (m, 8H); ^{13}C NMR (101 MHz, 298 K, MeCN-d3): 159.65, 152.26, 131.77, 130.95, 123.94, 106.97, 53.22, 52.12, 52.01, 39.60, 26.20, 26.05, 25.47 ppm; HR ESI-MS (MeCN) for $[C_{19}H_{25}N_5+H]^+$: m/z calcd: 324.2183; found: 324.2185; 0.6 ppm error.

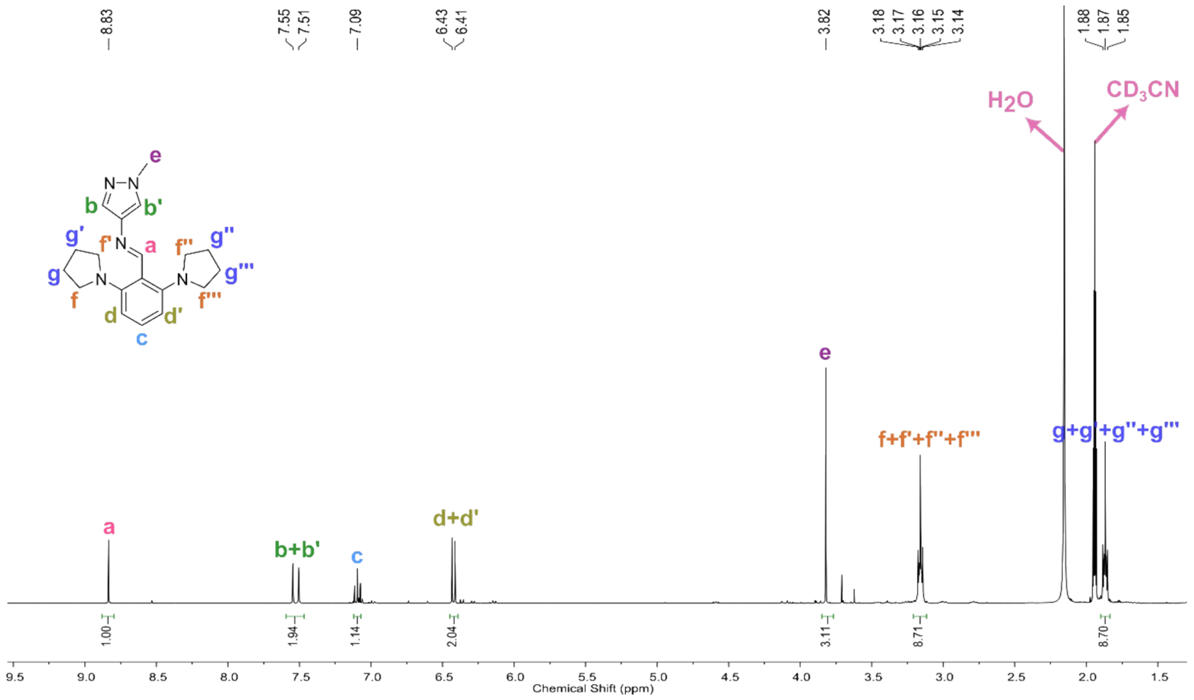

Figure S5. ^1H NMR (400 Hz, 298 K, MeCN-d3) of AIP **4**.

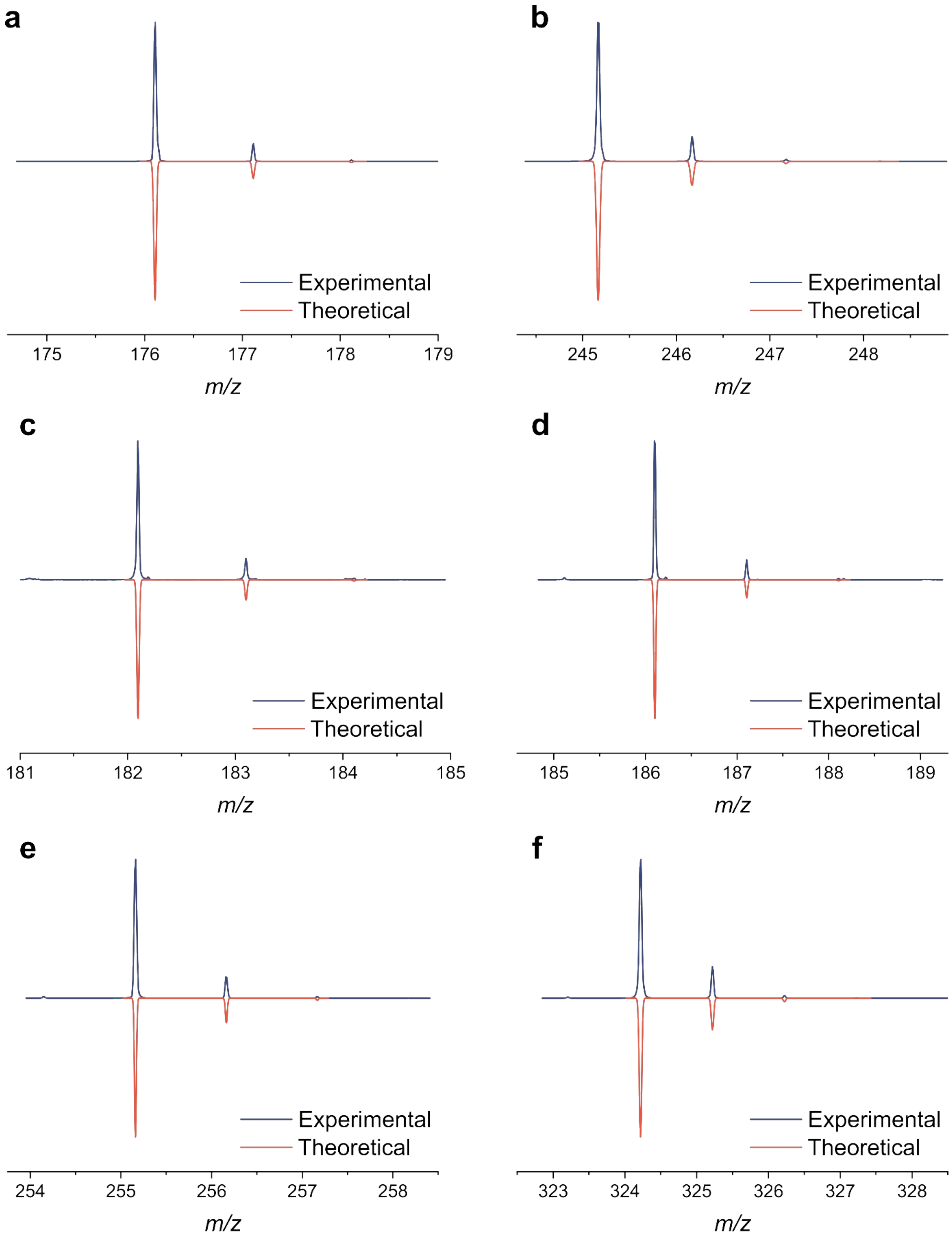

Figure S6. The HR-MS (ESI) of **a**) **S1**, **b**) **S2**, **c**) imine **1**, **d**) AIP **2**, **e**) AIP **3** and **f**) AIP **4**. The theoretical isotope pattern is shown in red and reflected in the x-axis. The experimental and theoretical traces are normalized for comparison.

4. Supporting and Characterisation Data

4.1 Figure of Merit Table

Table S3. An overview of the best performing photoswitchable aryl imines reported to date. The entries in this table were selected for their relatively long thermal half-life and PSS. Note that the short thermal half-lives of these photoswitches is anticipated to influence the maximum achievable %*Z*-isomer.

Structure	Maximum %*Z* achieved	$t_{1/2}$	π-π* (nm)	Reference
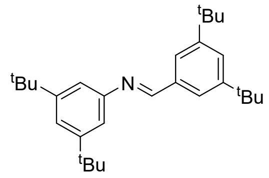	-	58 s (15 °C)	-	2
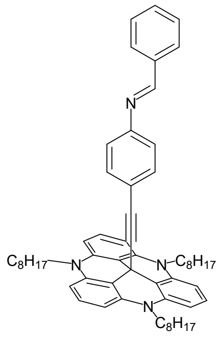	48%	0.6 s (20 °C)	334 nm	2
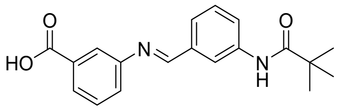	47%	<0.5 ms	*ca.* 320 nm	3
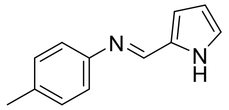	>6%	14 s (20 °C)	332 nm	4
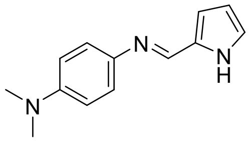	>40%	48 s (20 °C)	366 nm	4
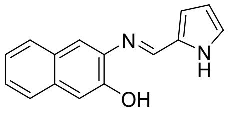	>3%	3.1 s (20 °C)	373 nm	4
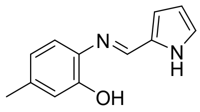	>5%	3.5 s (20 °C)	355 nm	4

4.2 Overview of Photoswitching Properties

This section contains additional information relating to the photoswitching properties of the AIP imines and reference compound **1**. The data presented in Table S5 was obtained by fitting the UV/vis absorption spectra of the *E*- and *Z*- isomers. Specifically, the peak analyser tool in OriginPro 2020 was used to fit the UV/vis spectra with the aim of deconvoluting overlapping transitions. The peaks were fitted to gaussians, and the results of the fit are presented in Table S5.

Table S4. Complete overview of the photostationary state (PSS), thermal half-life and quantum yield of photoisomerism data for the various wavelengths of irradiation used. The PSS values are obtained from UV/vis measurements performed in MeCN at 20°C. See Section for 4.5 for PSS values determined using ^{1}H NMR.

	Z-Isomer at Photostationary State (%)						$t_{1/2}$ (20 °C)	Quantum Yield *E*-to-*Z* (*Z*-to-*E*, %)				
	340 nm	365 nm	385 nm	405 nm	430 nm	470 nm		340 nm	365 nm	385 nm	405 nm	430 nm
**1**[a]	- (0[b], 1[c])	- (1[b], 14[c])	- (0[b], 2[c])	-	-	-	0.5 s	-	-	-	-	-
**2**[a]	- (13[b], 37[c])	- (21[b], 27[c])	- (5[b], 7[c])	-	-	-	12.5 s	3 (3)	-	-	-	-
**3**	35	66	82	95	90	55	22.1 min	2 (2)	2 (2)	3 (2)	3 (1)	4 (0)
**4**	38	68	79	95	99	94	19.2 h	1 (1)	1 (1)	1 (1)	1 (0)	1 (0)

[a] Note that the short $t_{1/2}$ values preclude the measurement of the %*Z* at the PSS under these conditions due to a significant amount of *Z*-to-*E* thermal reversion taking place during photoisomerism. [b] The maximum measurable %*Z* isomer recorded at 20°C. [c] The maximum measurable %*Z* isomer recorded at 0°C (Figure S15).

Table S5. Summary of the peak wavelengths of the fitted π-π* UV/vis absorption bands, and the differences in peak wavelength between the *E*- and *Z*-isomers. The measurements are performed in MeCN at 20°C. Details of the fitting are given at the start of this section.

	E-Isomer π-π*(nm)	*Z*-Isomer π-π*(nm)	Δπ-π* (*E*-*Z*, nm)
**1**	312	-	-
**2**	320	326	−6
**3**	370	345	26
**4**	363	310	53

4.3 Extinction Coefficient

The UV/vis spectra of all imines included in this work were measured using a Jasco V-770 UV/vis/NIR spectrophotometer at various concentrations. According to Beer-Lambert Law,[5] there exists a linear relationship between the absorbance and the concentration of the solution, which can be expressed as:

$$A = \epsilon l c$$

In this equation, "A" represents the absorbance in absorbance units (a.u.), "l" is the optical path length, which is typically the width of the cuvette used for the absorbance measurement (1 cm), and "c" is the molar concentration in moles per liter (M). From the absorption spectra (Figure S7), the molar extinction coefficient (ε) at the specific wavelength (λ_{max}) can be determined by fitting a linear calibration curve of the absorbance versus concentration.

Table S6. The wavelengths of maximum absorbance (λ_{max}) and the extinction coefficients at λ_{max} of each switch as the *E*-isomer. These values are obtained directly from the recorded data without deconvolution of overlapping bands.

	**λ_{max}** (nm)	**Extinction Coefficient at λ_{max}** (M^{-1} cm^{-1})
**1**	310	7900
**2**	315	14200
**3**	370	8300
**4**	363	6300

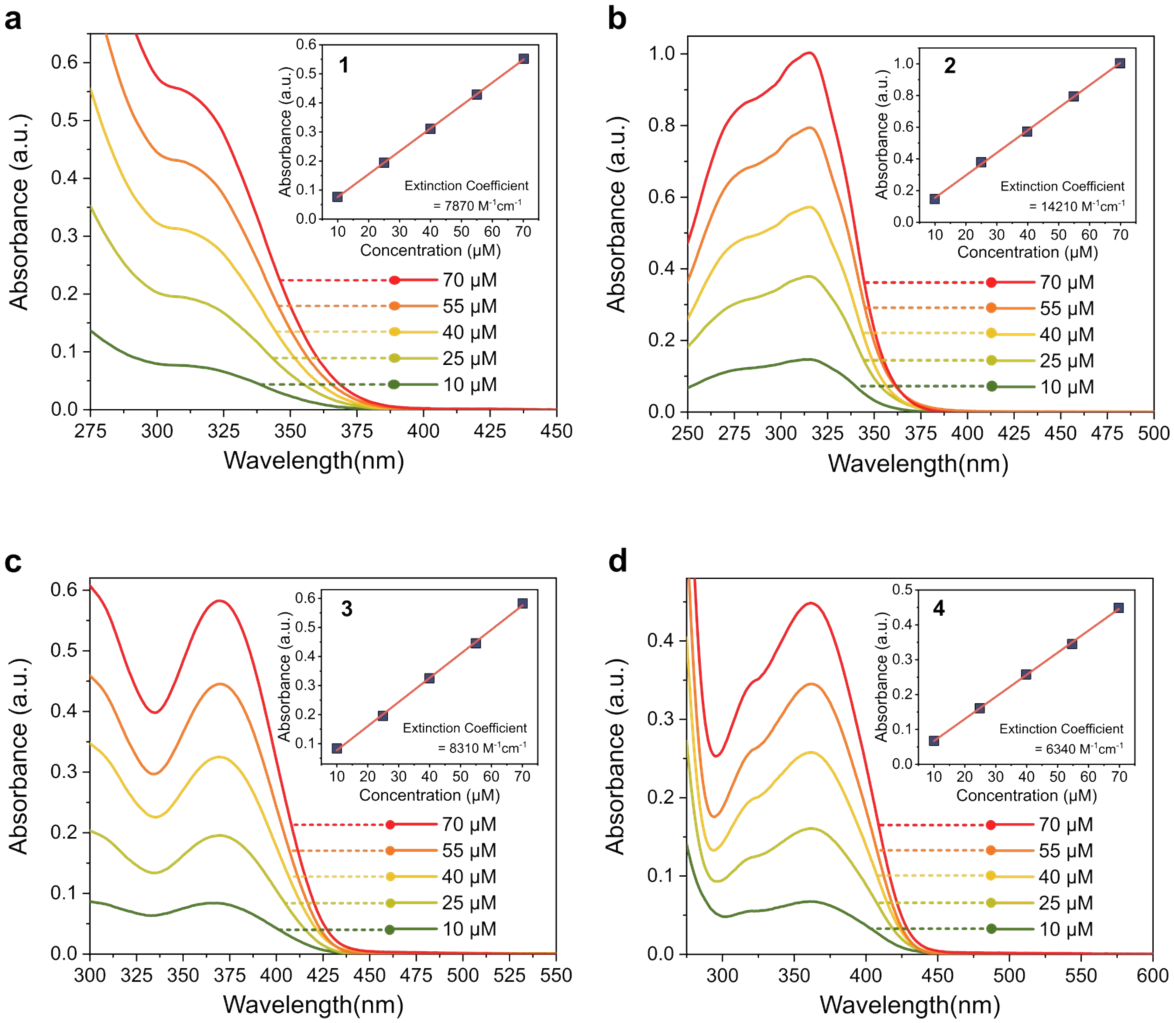

Figure S7. The UV/vis absorption spectra of imines **1**, **2**, **3**, and **4** were measured at 20 °C in acetonitrile, each at different concentrations. The calibration curves, shown in the top right corner of each figure, were plotted at the wavelength of λ_{max} to determine the slopes, which represent the extinction coefficients.

4.4 Thermodynamic Properties

The kinetics of all imine switches included in this work were investigated using the diode array setup (Section 1.2.1 of the Supporting Information). These measurements were conducted in the solution state using acetonitrile as the solvent, and a range of temperatures was employed for linear Eyring plots. The isomerization from *E*-isomers to *Z*-isomers was induced by irradiation with 365 nm LED light. Assuming first-order reactions, the rate constants (k) for the thermodynamic switching process from *Z*- to *E*-state were determined by fitting the absorbance changes over time to an exponential (Figures S8-S11), and the thermal half-lives ($t_{1/2}$) were calculated based on this rate constants.

Utilizing the linearized form of the Eyring equation presented below:[6]

$$ln\frac{k}{T} = \frac{-\Delta H^{\ddagger}}{R} \cdot \frac{1}{T} + ln\frac{k_B}{h} + \frac{\Delta S^{\ddagger}}{R}$$

The values for enthalpy of activation ($\Delta H^{\ddagger}$) and entropy of activation ($\Delta S^{\ddagger}$) in Table S7 can be determined from the kinetic data at different temperatures by linear fitting (Figure S12). Gibbs energy of activation (listed in Table S7) can be calculated using the Gibbs function:

$$\Delta G^{\ddagger} = \Delta H^{\ddagger} - T\Delta S^{\ddagger}$$

Table S7. The summary of activation parameters including thermal half-life (at 20 °C), enthalpy of activation ($\Delta H^{\ddagger}$), entropy of activation ($\Delta S^{\ddagger}$), and Gibbs energy of activation ($\Delta G^{\ddagger}$) data of imines discussed in this work. In this table, the Gibbs energy of activation corresponds to a temperature of 295K.

	$t_{1/2}$	**ΔH‡** ($kJ \cdot mol^{-1}$)	**ΔS‡** ($J \cdot K^{-1} \cdot mol^{-1}$)	**ΔG‡** ($kJ \cdot mol^{-1}$)
**1**	0.5 s	69.23	−6.35	71.15
**2**	12.5 s	79.34	1.98	78.75
**3**	22.1 min	86.27	-13.25	90.18
**4**	19.2 h	105.08	17.56	99.90

4.4.1 Thermal Half-Life Data

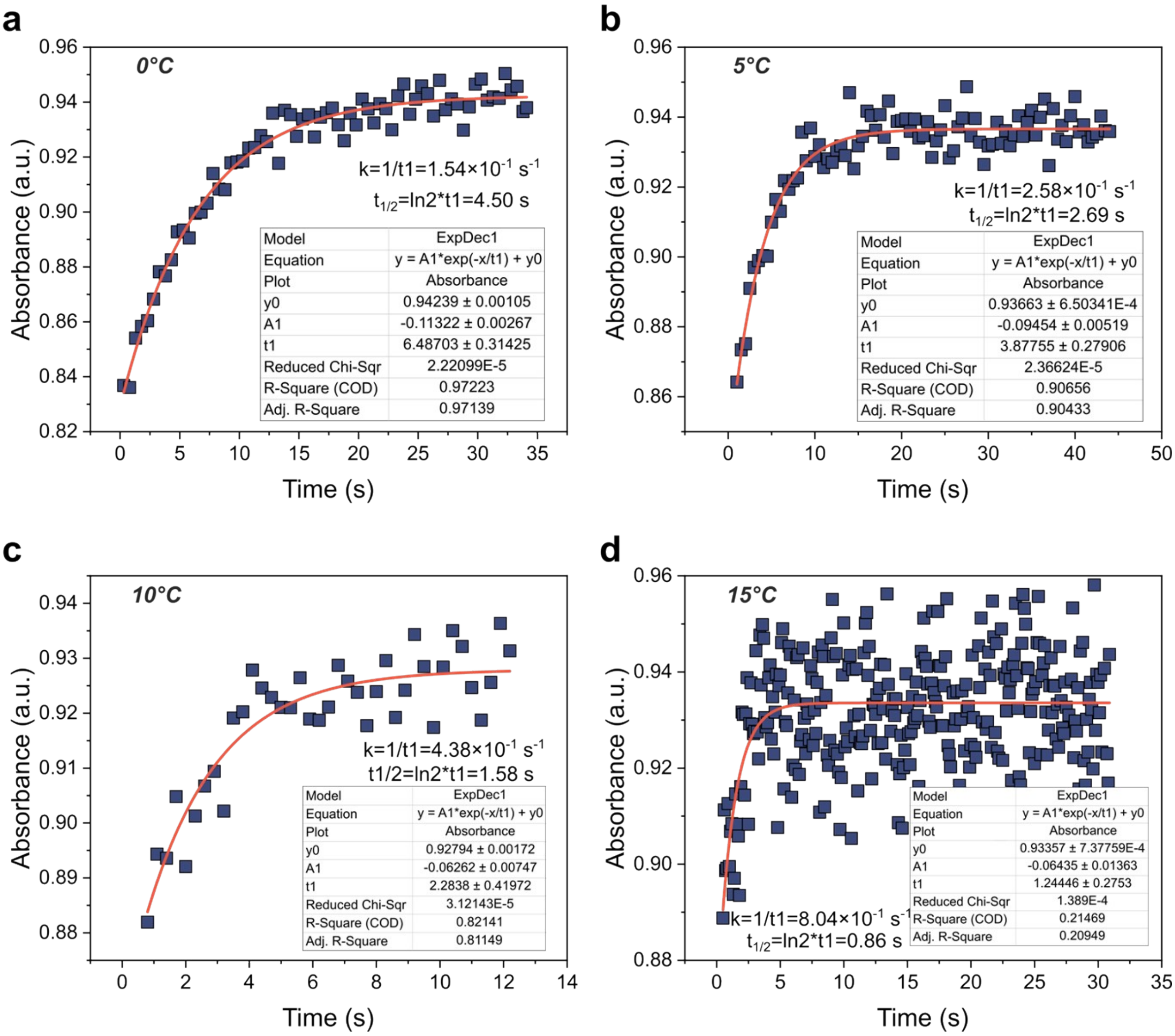

Figure S8. The change in absorbance of **1** at the λ_{max} of the *E*-isomer over time at **a**) 0 °C, **b**) 5 °C, **c**) 10 °C, **d**) 15 °C, with the increase in absorbance relating to the thermal isomerization of the *Z*-isomer to the *E*-isomer. All samples were irradiated under 365 nm LED for 5 minutes in acetonitrile before the measurements started. The exponential fittings were applied to determine the rate constants and thus thermal half-lives. Note that the scatter in plot d is attributed to the short half-life.

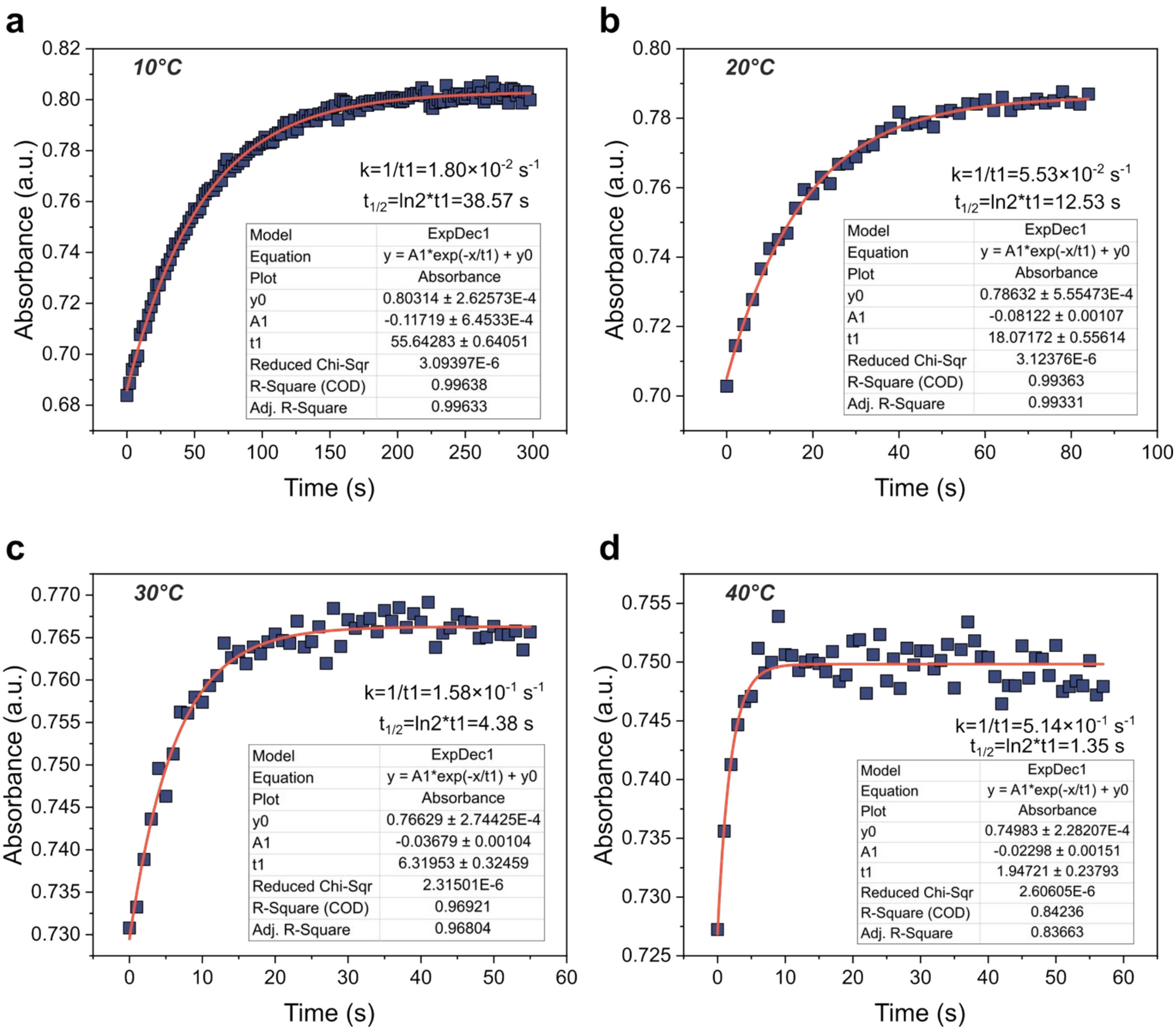

Figure S9. The change in absorbance of **2** at the λ_{max} of the *E*-isomer over time at **a**) 10 °C, **b**) 20 °C, **c**) 30 °C, **d**) 40 °C, with the increase in absorbance relating to the thermal isomerization of the *Z*-isomer to the *E*-isomer. All samples were irradiated under 365 nm LED for 5 minutes in acetonitrile before the measurements started. The exponential fittings were applied to determine the rate constants and thus thermal half-lives.

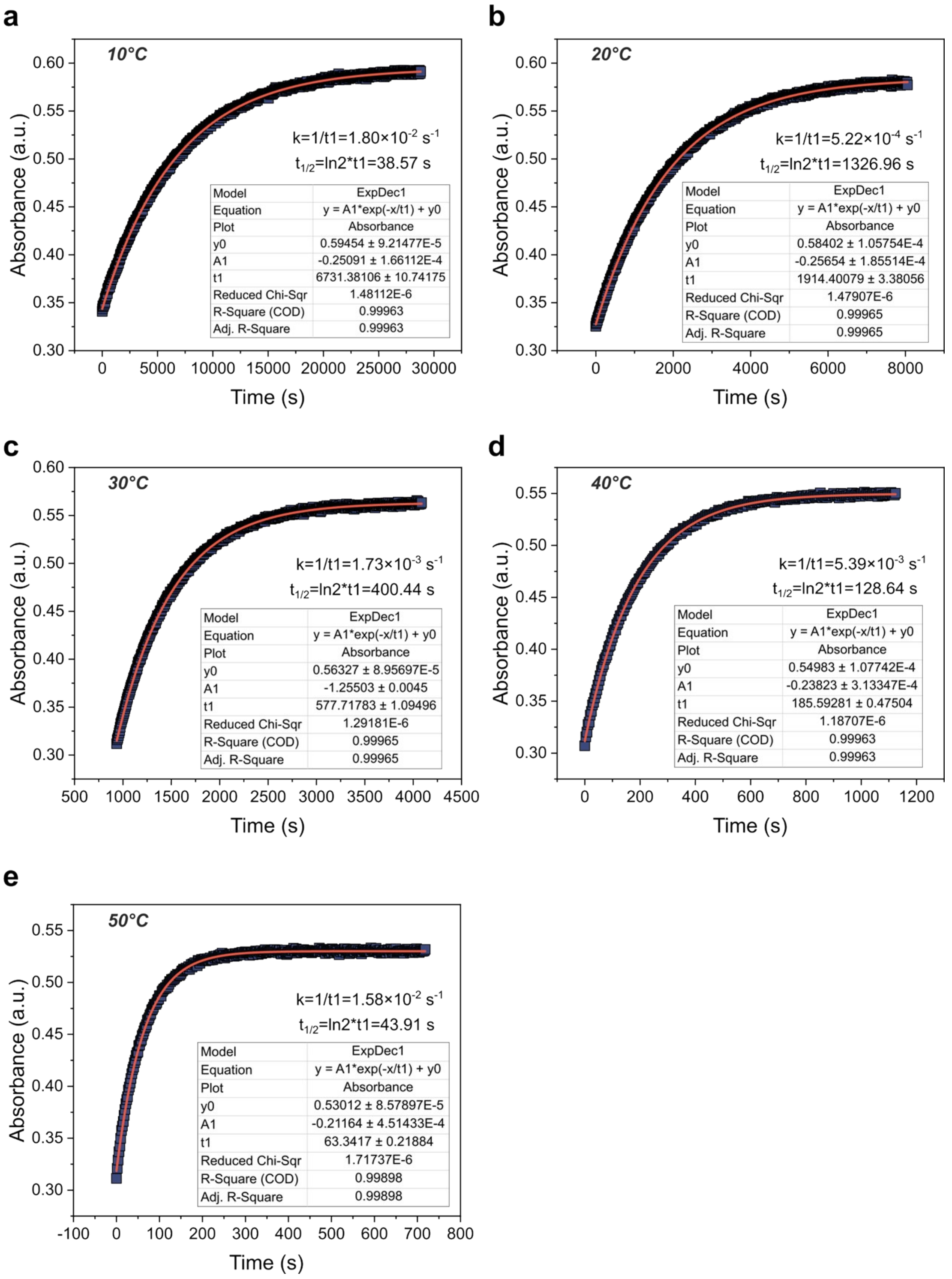

Figure S10. The change in absorbance of **3** at the λ_{max} of the *E*-isomer over time at **a**) 10 °C, **b**) 20 °C, **c**) 30 °C, **d**) 40 °C, **e**) 50 °C, with the increase in absorbance relating to the thermal isomerization of the *Z*-isomer to the *E*-isomer. All samples were irradiated under 365 nm LED for 5 minutes in acetonitrile before the measurements started. The exponential fittings were applied to determine the rate constants and thus thermal half-lives.

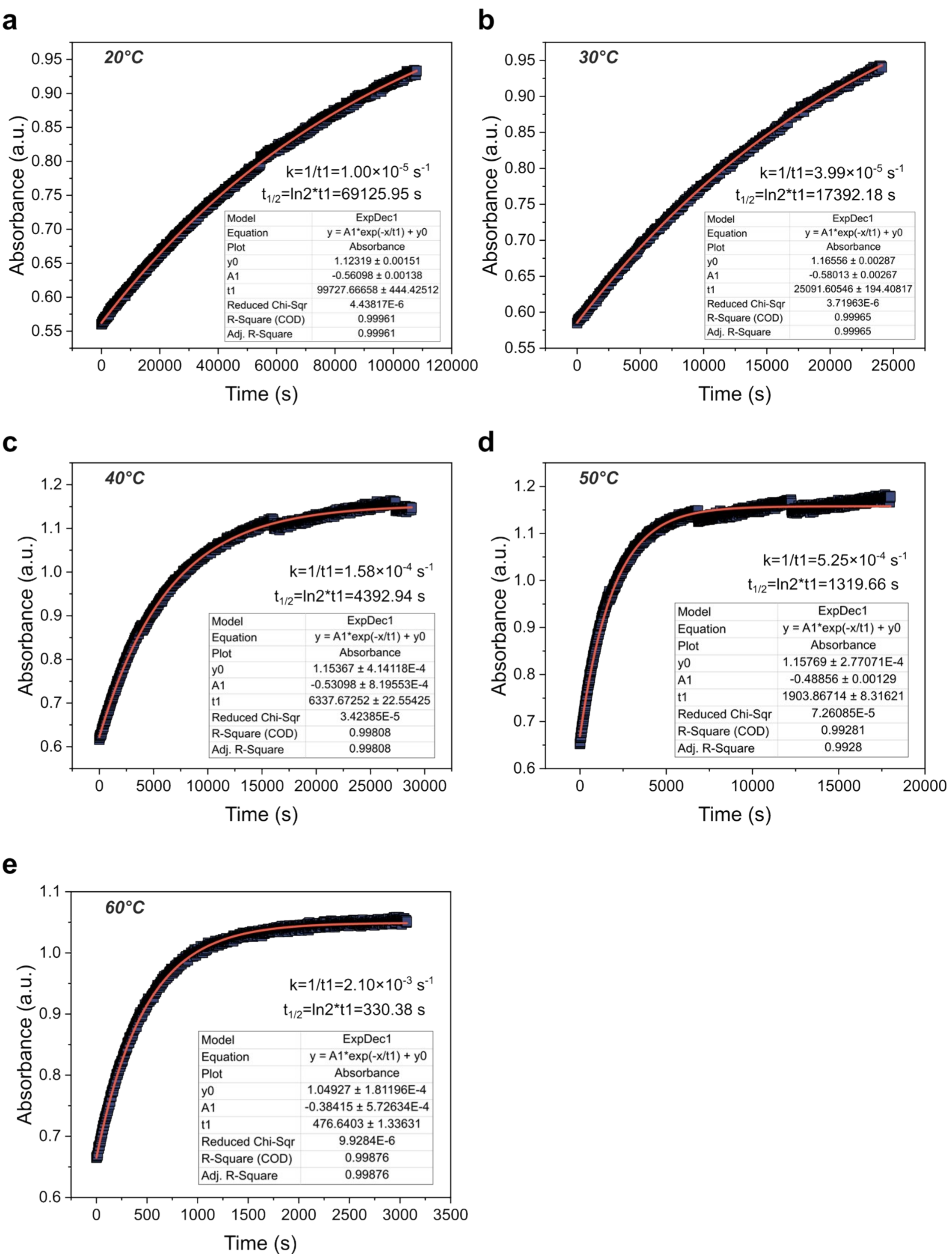

Figure S11. The change in absorbance of **4** at the λ_{max} of the *E*-isomer over time at **a**) 20 °C, **b**) 30 °C, **c**) 40 °C, **d**) 50 °C, **e**) 60 °C, with the increase in absorbance relating to the thermal isomerization of the *Z*-isomer to the *E*-isomer. All samples were irradiated under 365 nm LED for 5 minutes in acetonitrile before the measurements started. The exponential fittings were applied to determine the rate constants and thus thermal half-lives.

4.4.2 Fitting of Rate Constants to the Eyring Equation

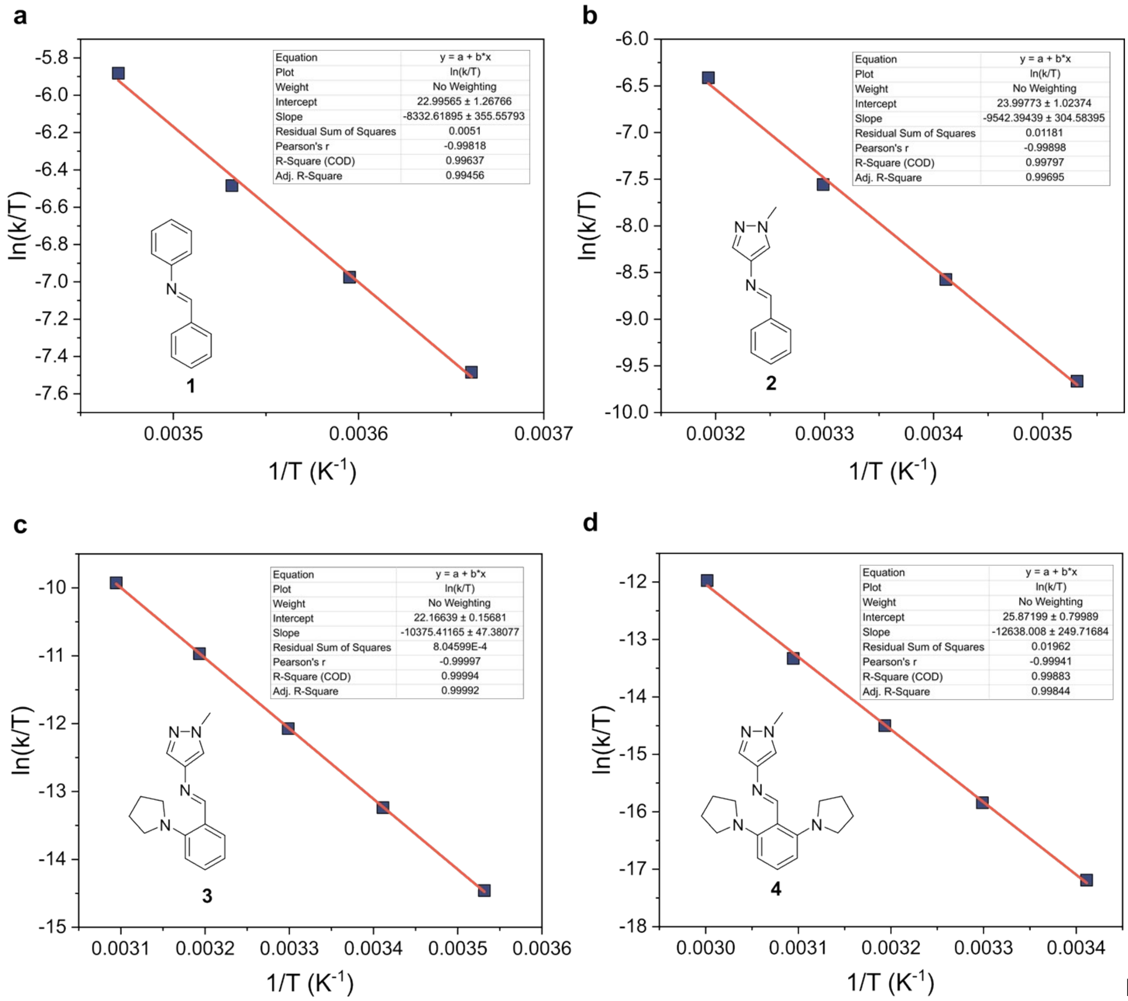

Figure S12. The Eyring plots of **1** (70 µM in MeCN), **2** (55 µM in MeCN), **3** (70 µM in MeCN) and **4**(170 µM in MeCN) were generated using the rate constants calculated at different temperatures. The data exhibited linearity with R^2 values exceeding 0.99. The fitted parameters were used to estimate the enthalpy of activation ($\Delta H^{\ddagger}$), entropy of activation ($\Delta S^{\ddagger}$), and Gibbs energy of activation ($\Delta G^{\ddagger}$) listed in Table S7.

4.5 Photostationary States (PSS)

Irradiation for PSS measurements was conducted using the diode array setup (Section 1.2.1) with LED lights ranging from 340 nm to 470 nm, as detailed in Table S1.

The proportions of the *Z*-isomers at the PSS reported in Table 1 and Table S4 were determined by UV/vis spectroscopy at 20 °C, using spectroscopy grade acetonitrile as the solvent. The calculations of PSS were performed according to Fischer's approach,[7] by analysing the absorption spectra of the pure *E*-isomer before irradiation and after photo-equilibration at two different wavelengths. We specifically chose wavelengths where the quantum yield of the *E*-to-*Z* and *Z*-to-*E* isomers were identical, which was the case for 340 nm and 365 nm irradiation, a crucial assumption in Fischer's approach. From this, the UV/vis of the pure *Z*-isomer was predicted (see below). We then a linearly interpolated between the dark (pure *E*-isomer) and predicted *Z*-isomer to determine the PSS at other irradiation wavelengths.

The absorption spectra of *Z*-isomers can be estimated by the relationship between the dark spectra (Pure *E*), the PSS spectra and the PSS quantity, as presented below:

$$Estimated\ Pure\ Z = (Spectra\ of\ pure\ E) + \frac{(PSS\ spectra) - (Pure\ E\ spectra)}{(PSS\ quantity)}$$

Additionally, for photoswitches with extended thermal half-lives (≥20 minutes), ^{1}H NMR measurements were employed to ascertain the isomer ratios, as shown in Figure 3b and Figure S14. These results corroborated the values obtained from UV/vis spectroscopy. The PSS distributions are shown in Figure S13 and are summarized in Table S4.

It is important to note that our setup, shown schematically in Figure S1, facilitates the rapid acquisition of the UV/vis spectra: 50 ms integration time, average of 10 spectra, resulting in a spectrum being recorded every 0.5 s. The combination of the high intensity of irradiation, rapid spectral acquisition, and temperature control limits the amount of time between irradiation and spectral acquisition, providing a reliable measurement of the PSS at the given irradiation wavelength and temperature. Note that the underlying competing *Z*-to-*E* thermal relaxation process does influence the measured %*Z* isomer of the photoswitches with a short $t_{1/2}$, as indicated in Figure S15. Thus, we report the maximum measured %*Z* isomer for imines **1** and **2** since the PSS is not reached under these conditions. The experimentally measured quantity of *Z*% is an important descriptor when selecting photoswitches for a particular application.

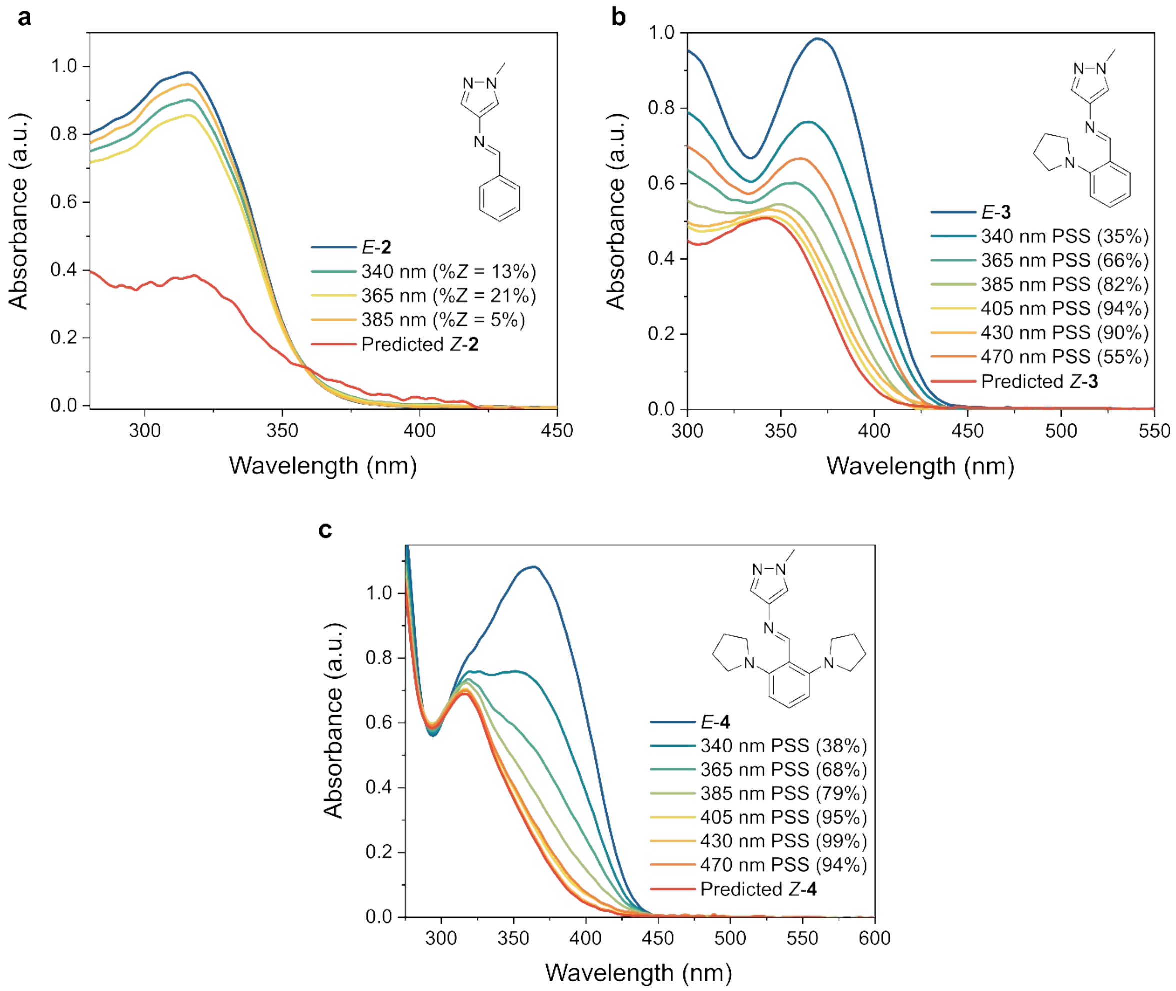

Figure S13. The UV/vis spectra of **2** (70 μM in MeCN), **3** (120 μM in MeCN) and **4** (170 μM in MeCN) under different irradiation conditions, alongside the predicted *Z*-isomer spectra. For switches **3** and **4**, the proportions of *Z*-isomers under different wavelength irradiations were quantified as PSS values, with the *E*-isomer set at 0% and the predicted *Z*-isomer at 100%.

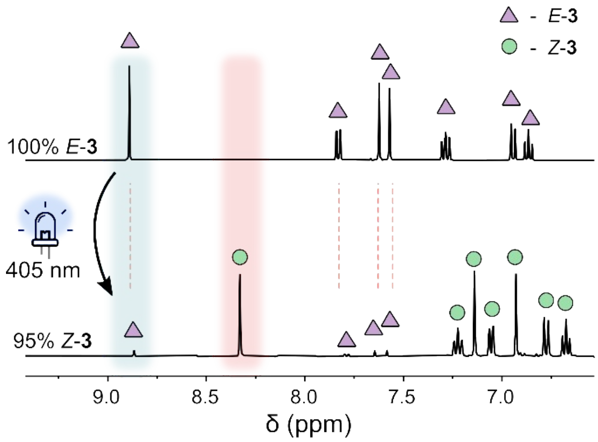

Figure S14. ^{1}H NMR spectra (400 MHz, MeCN-d3) of **3** before irradiation (above) and after irradiation at 405 nm LED (below). The *Z*-isomer proportion was determined by integration of labelled signals to determine PSS at the specified wavelength. The sample was irradiated in NMR tubes until no further change was observed in the distribution of the *E*/*Z* isomers in the ^{1}H NMR spectra.

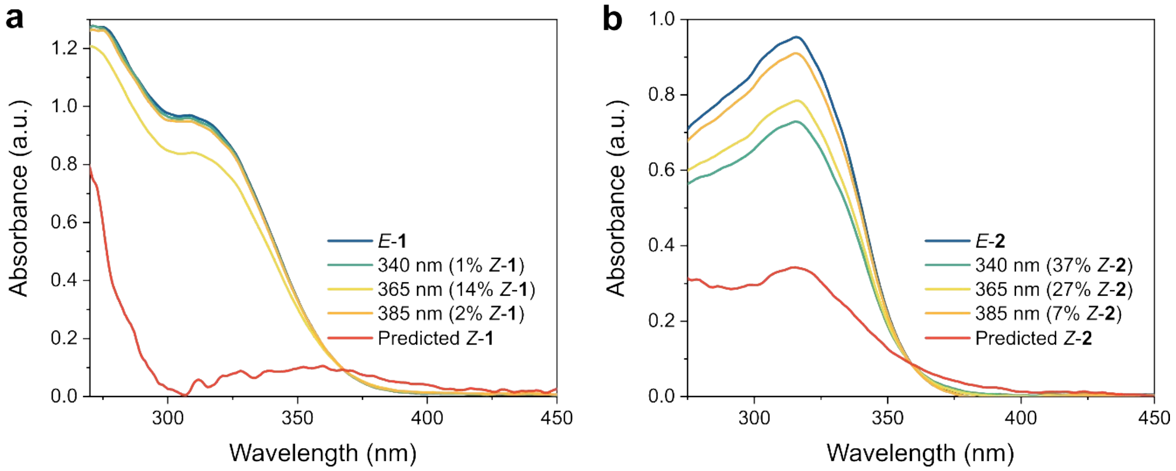

Figure S15. The UV/vis spectra of **a**) **1** (130 µM in MeCN) and **b**) **2** (70 µM in MeCN) under different irradiation conditions, alongside the predicted *Z*-isomer spectra, measured at 0°C. Note that a greater proportion of the *Z*-isomer is obtained at 0°C compared to 20°C, attributed to a decreased rate of *Z*-to-*E* thermal isomerization.

4.6 Fatigue Resistance

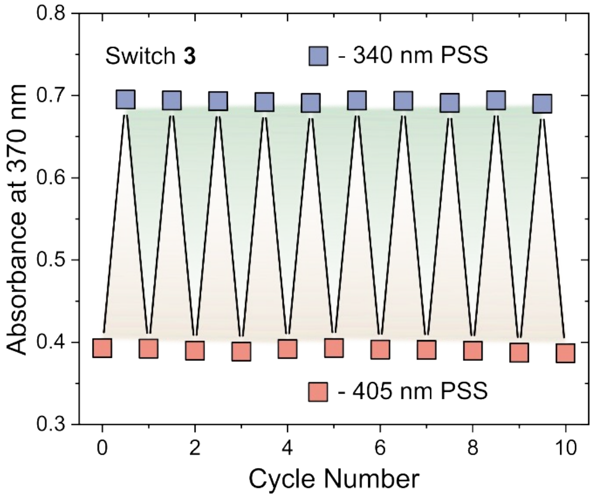

Figure S16. Plot showing the fatigue resistance of AIP **3** to repeated photoisomerism with 340 nm and 405 nm irradiation. No signs of degradation are observed over 10 switching cycles.

4.7 Quantum Yields

The ferrioxalate method, originally introduced by Parker and Hatchard,[8] was employed for photon flux measurements spanning wavelengths from 340 nm to 430 nm. The method is based on the photochemical degradation of ferrioxalate to Fe^{2+}:

$$Fe(C_2O_4)_3^{3-}\ h\upsilon \rightarrow Fe^{2+}\ C_2O_4^{\cdot-} + 2C_2O_4^{2-}$$

$$Fe(C_2O_4)_3^{3-} + C_2O_4^{\cdot-} \rightarrow Fe^{2+} + 2CO_2 + 3C_2O_4^{2-}$$

According to the reported protocol,[9] 2 mL of 30 mM ferrioxalate in 0.2 N H_2SO_4 was irradiated using LED lights at different wavelengths for 30-150 s (diode array setup, Section 1.2.1). Subsequently, 0.5 mL of irradiated solution was mixed with 1 mL buffer (1.2 M NaAc + 0.72 N H_2SO_4) and 2 mL phenanthroline (6 mM), diluted to 25 mL and allowed to react in the dark for ≥1 h. Absorbance of the resulting tris-phenanthroline complex was measured at 510 nm (ε_{510nm}=11100 M^{-1} cm^{-1}), as shown in Figure S17. The photon flux was determined by fitting the absorbance data using the software provided by Stranius and Börjesson.[9] Specifically, the change in absorbance over time is fitted to the equation below which takes into account the thermal relaxation process (*Z* to *E*):

$$\frac{d[A]}{dt} = -\frac{\varphi_A \cdot I \cdot \beta_A(t)}{N_a \cdot V} + \frac{\varphi_B \cdot I \cdot \beta_B(t)}{N_a \cdot V} + k_{t,B\rightarrow A}[B]$$

Where φ is the quantum yield of photoisomerization of a given species, I is the photon flux, β is the fraction of photons absorbed by a given species, V is the volume of sample, N_a is Avogadro's numbers, k_t is the rate of the spontaneous back conversion.

All imine switch samples were prepared in acetonitrile at room temperature. The irradiation and measurements were performed in the diode array simultaneously, with the same irradiation conditions as the previous calibration process: **a**, 340 nm (500 mA, 90%), **b**, 365 nm (200 mA, 20%), **c**, 385 nm (200 mA, 20%), **d**, 405 nm (200 mA, 20%), **e**, 430 nm (200 mA, 20%), respectively.

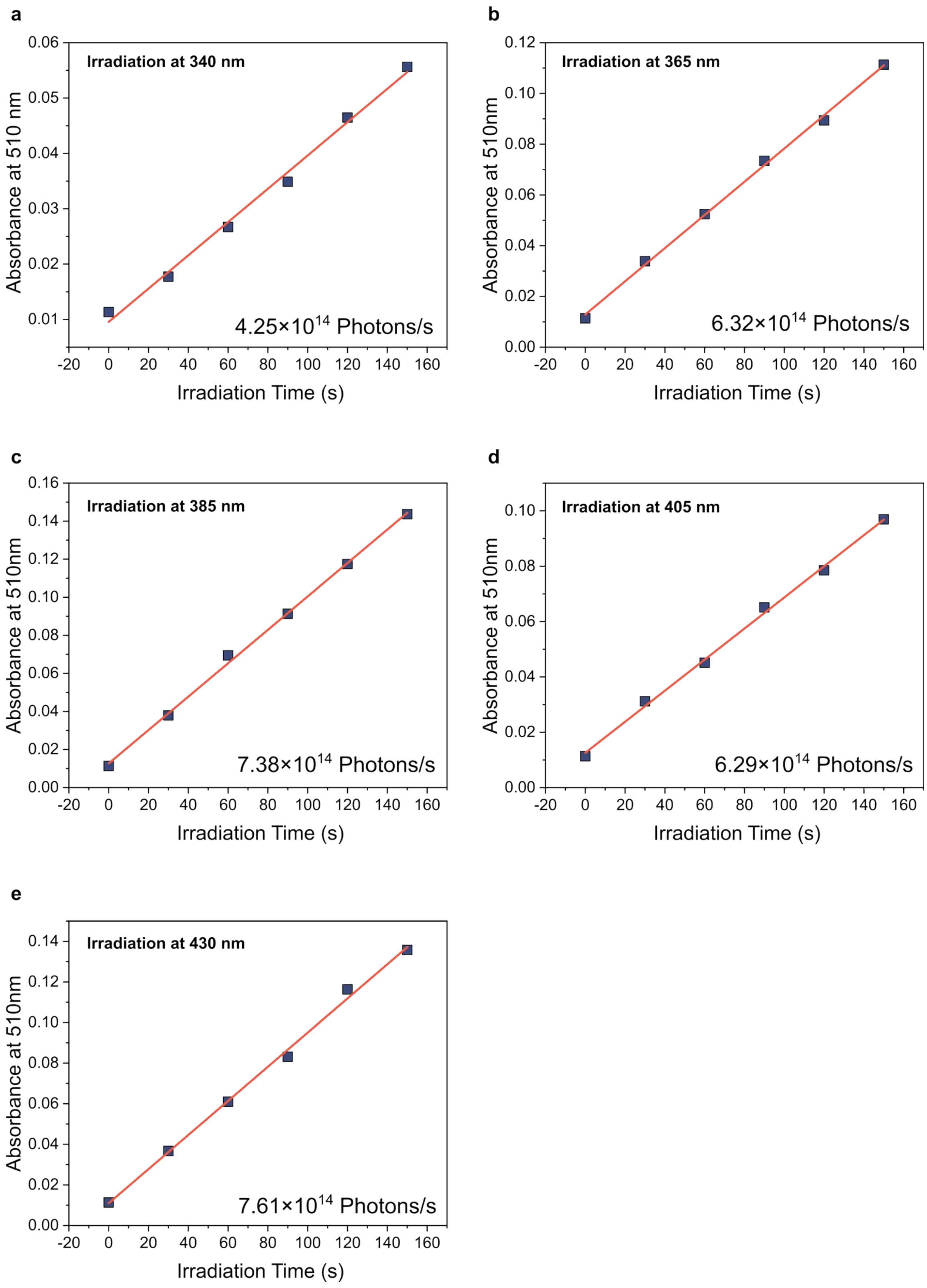

Figure S17. Determination of photon flux from **a**, 340 nm (500 mA, 90%), **b**, 365 nm (200 mA, 20%), **c**, 385 nm (200 mA, 20%), **d**, 405 nm (200 mA, 20%), **e**, 430 nm (200 mA, 20%) LED. The plots of **a**-**e** follow the absorbance of Fe(II) phenanthroline complex as a function of irradiation time.

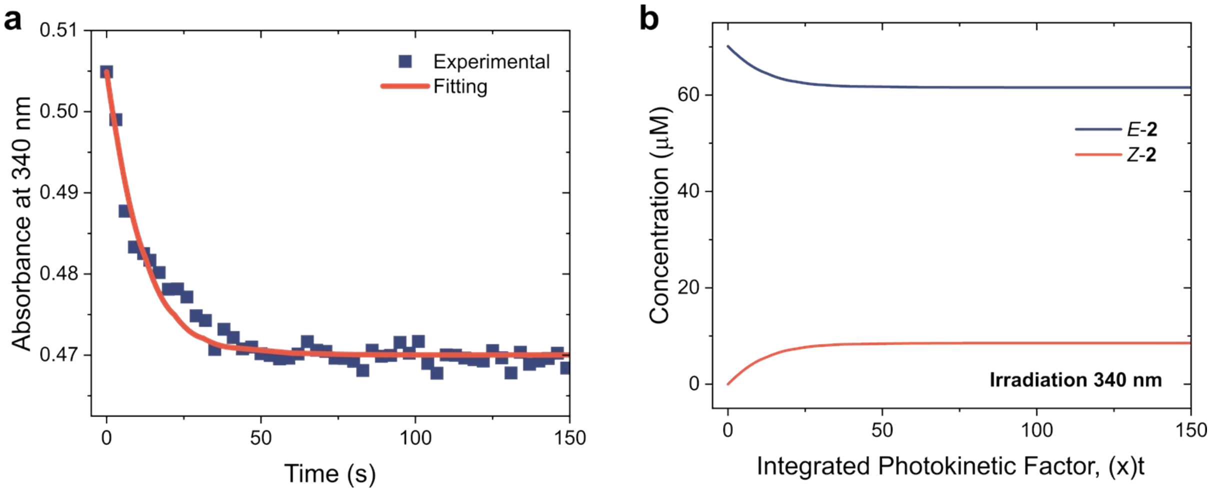

Figure S18. a, The plot of the change in absorbance at 340 nm as a function of irradiation time with 340 nm light for AIP **2**. The fitted trace is shown in red. **b**, The integrated photokinetic factor, (x)t plotted against the concentration of *E* and *Z* Isomers of **2** using 340 nm (500 mA, 90%) light. The calculated quantum yields were determined using a previously published method,[9] the results for all imines can be found in Table S4.

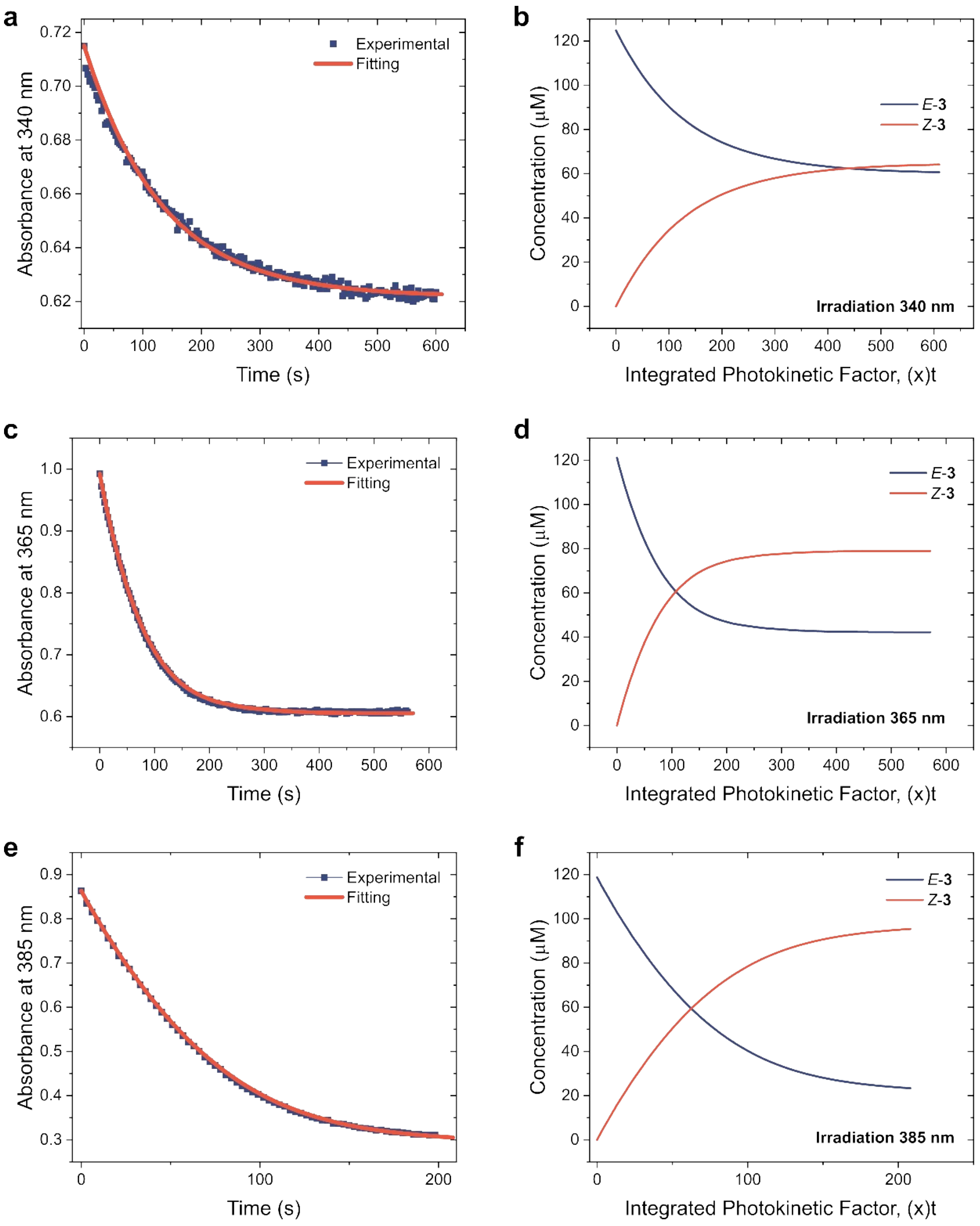

Figure S19. The plot of the change in absorbance as a function of irradiation time with **a**, 340 nm, **c**, 365 nm, **e**, 385 nm light for AIP **3**. The fitted trace is shown in red. The integrated photokinetic factor, (x)t plotted against the concentration of *E* and *Z* Isomers of **3** using **b**, 340 nm (500 mA, 90%), **d**, 365 nm (200 mA, 20%), **e**, 385 nm (200 mA, 20%) light. The calculated quantum yields were determined using a previously published method,[9] the results for all imines can be found in Table S4.

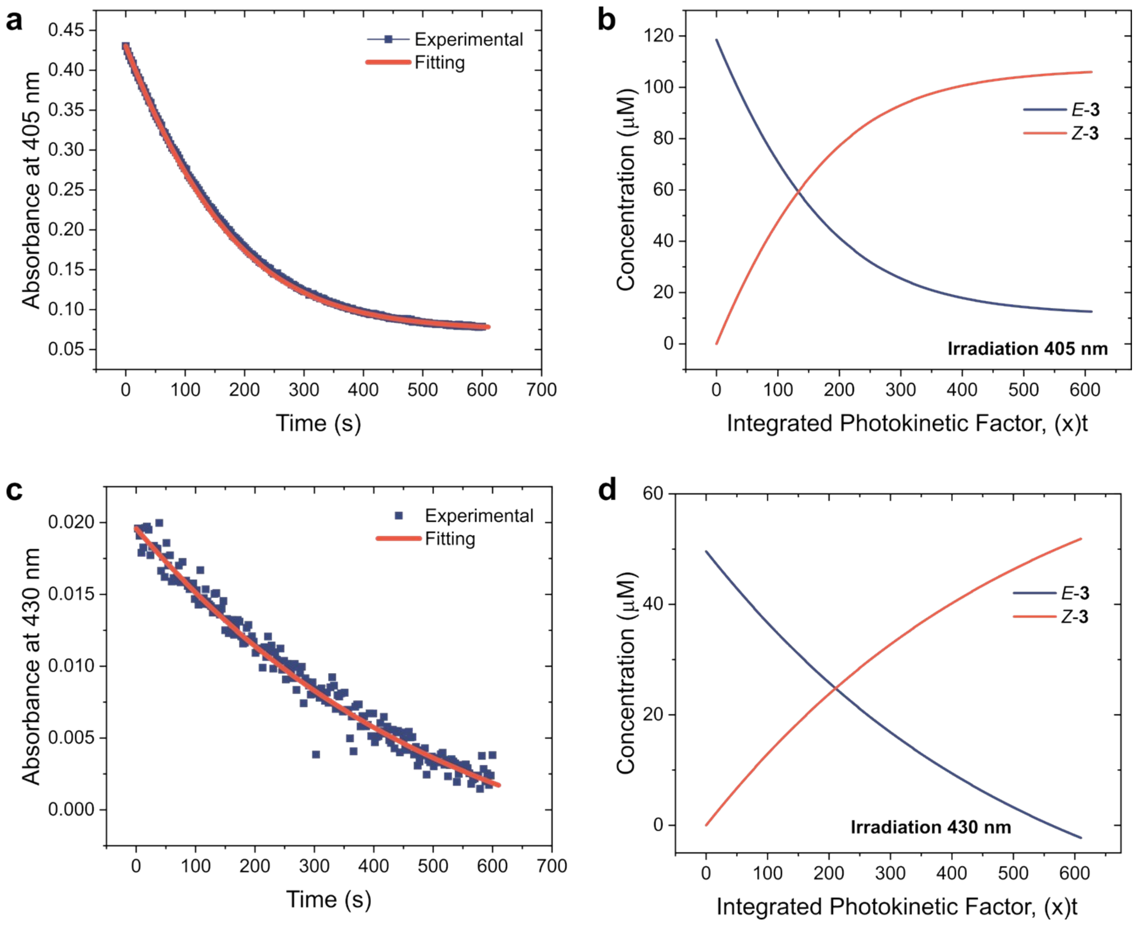

Figure S20. The plot of the change in absorbance as a function of irradiation time with **a**, 405 nm, **c**, 430 nm light for AIP **3**. The fitted trace is shown in red. The integrated photokinetic factor, (x)t plotted against the concentration of *E* and *Z* Isomers of **3** using **b**, 405 nm (200 mA, 20%), **d**, 430 nm (200 mA, 20%) light. The calculated quantum yields were determined using a previously published method,[9] the results for all imines can be found in Table S4.

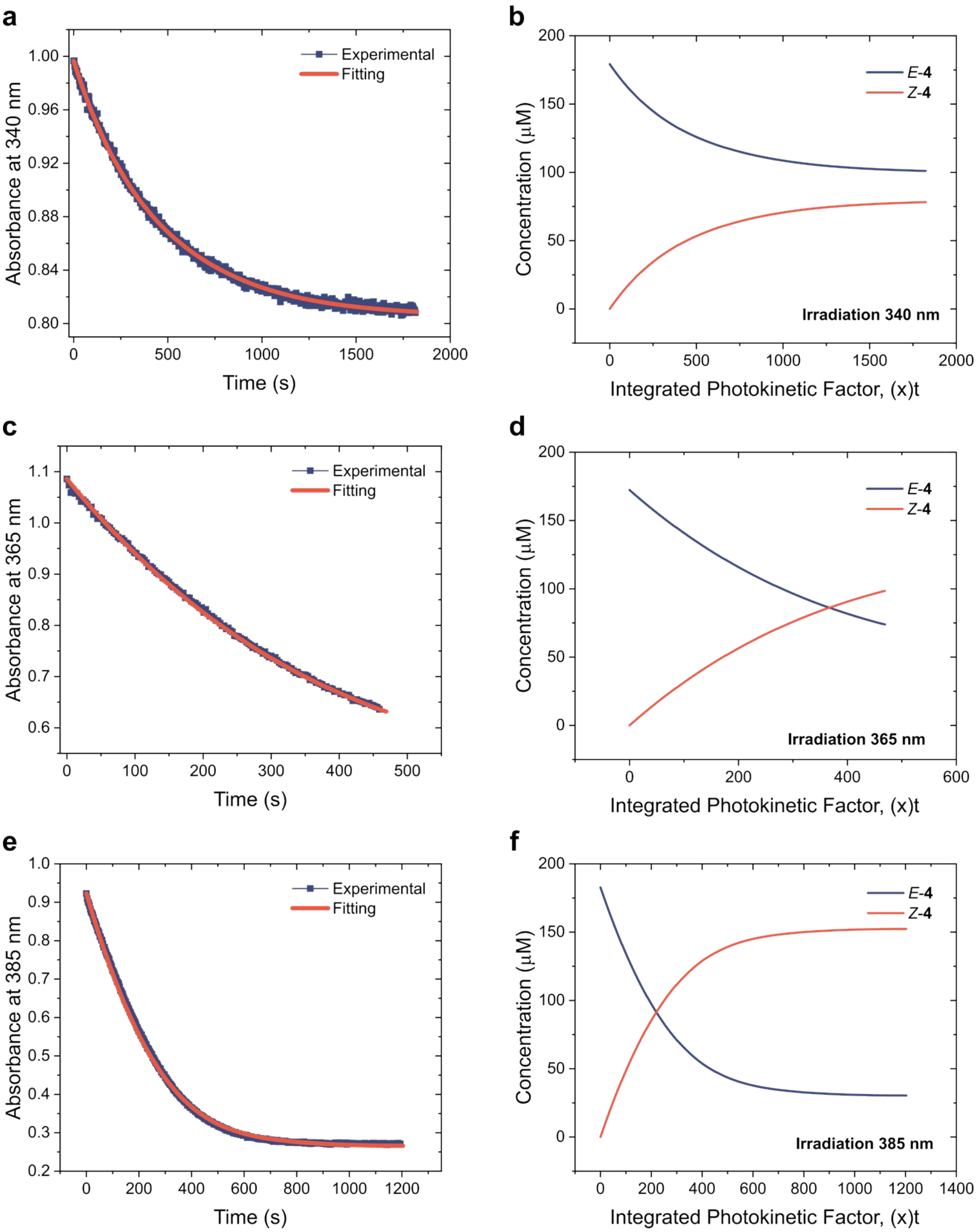

Figure S21. The plot of the change in absorbance as a function of irradiation time with **a**, 340 nm, **c**, 365 nm, **e**, 385 nm light for AIP **4**. The fitted trace is shown in red. The integrated photokinetic factor, (x)t plotted against the concentration of *E* and *Z* Isomers of **4** using **b**, 340 nm (500 mA, 90%), **d**, 365 nm (200 mA, 20%), **e**, 385 nm (200 mA, 20%) light. The calculated quantum yields were determined using a previously published method,[9] the results for all imines can be found in Table S4.

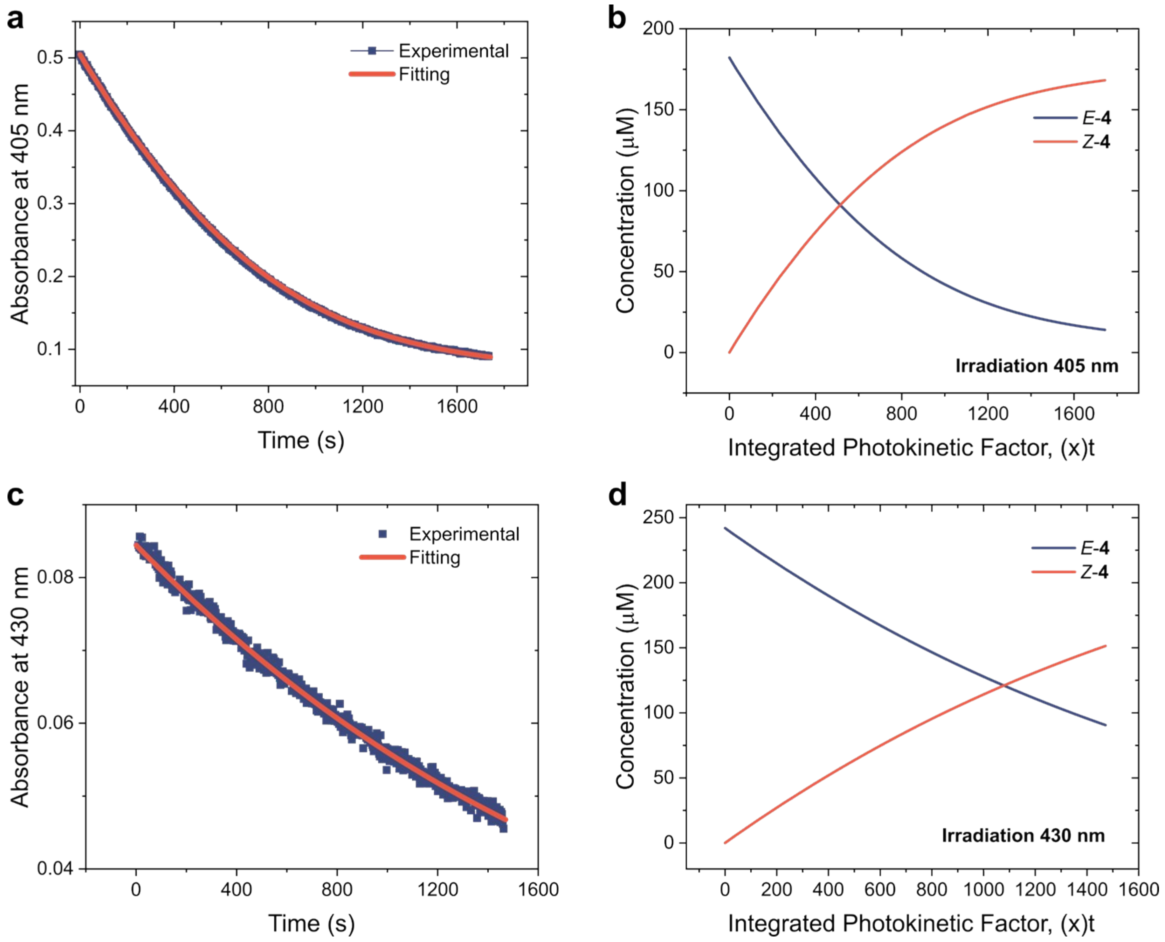

Figure S22. The plot of the change in absorbance as a function of irradiation time with **a**, 405 nm, **c**, 430 nm light for AIP **4**. The fitted trace is shown in red. The integrated photokinetic factor, (x)t plotted against the concentration of *E* and *Z* Isomers of **4** using **b**, 405 nm (200 mA, 20%), **d**, 430 nm (200 mA, 20%) light. The calculated quantum yields were determined using a previously published method,[9] the results for all imines can be found in Table S4.

4.8 Influence of Solvent

The impact of the solvent on the photoswitching properties of AIP **4** was investigated. The results are presented below in Table S8 and Figures S23 and S24.

Table S8. Summary of the photoswitching properties of AIP **4** in different solvents. The PSS values reported here were obtained from UV/vis measurements performed at 20°C.

Solvent	(E_T^N)[a]	%*Z* at PSS						*E*-Isomer π-π*	$t_{1/2}$ (h)
		340 nm	365 nm	385 nm	405 nm	430 nm	470 nm	λ_{max} (nm)	(20°C)
Toluene	0.099	35	70	85	97	99	95	365	8.3
1,4-Dioxane	0.164	40	66	80	92	96	92	364	12.4
THF	0.207	43	68	87	98	99	96	363	8.4
DCM	0.309	38	62	81	96	98	92	368	3.2
DMSO	0.444	36	65	82	99	100	99	366	14.7
MeCN	0.460	38	68	79	95	99	94	363	19.2

[a] (E_T^N) is an empirical solvent parameter which is used here as a measure of solvent polarity.[10]

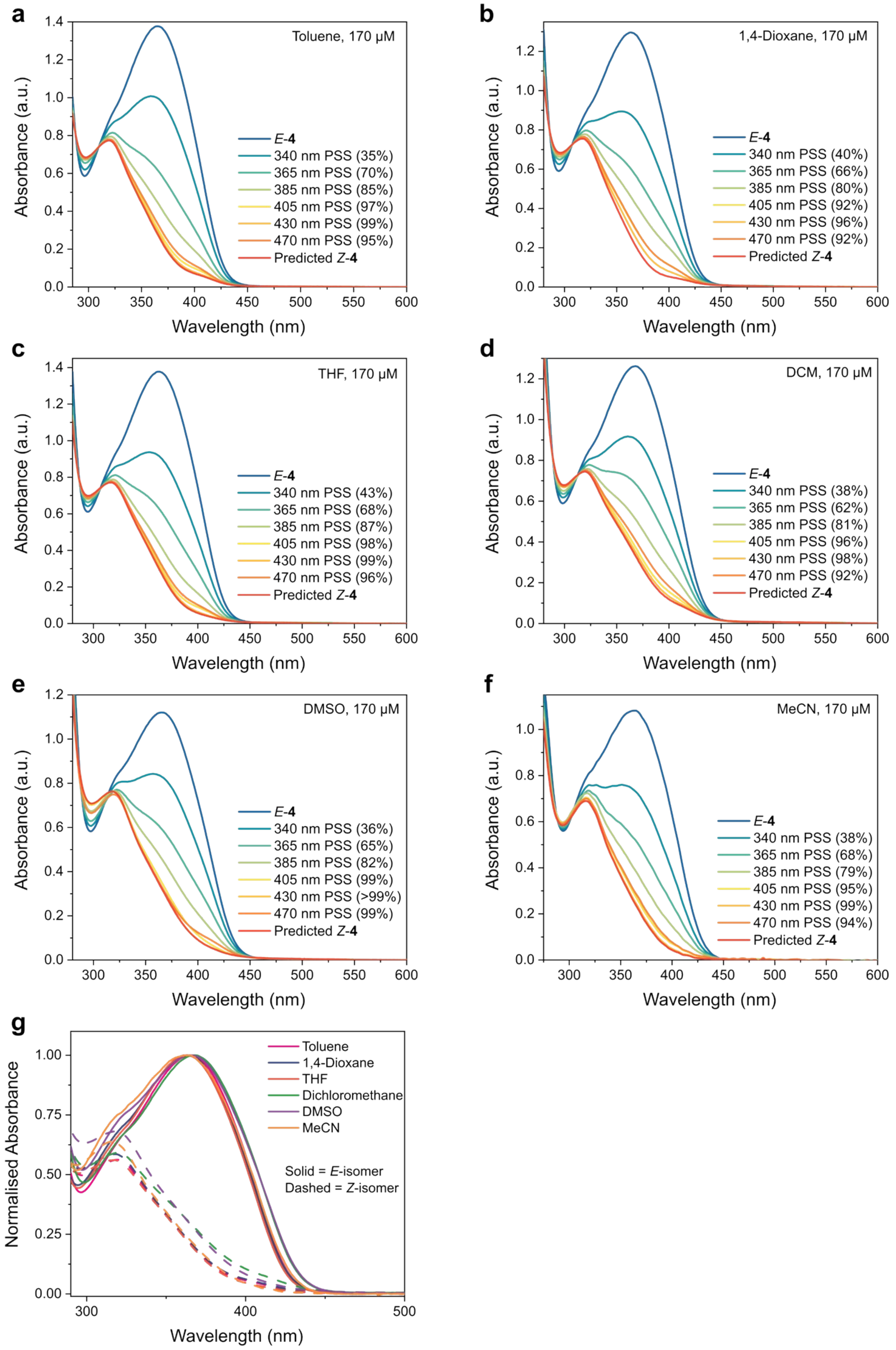

Figure S23. a-f, The UV/vis spectra of **4** (170 μM) in various solvents under different irradiation conditions, alongside the predicted *Z*-isomer spectra. The proportions of *Z*-isomers under different wavelength irradiations were quantified as PSS values, with the *E*-isomer set at 0% and the predicted *Z*-isomer at 100%. **g**, Plot of the spectra of the *E*- (solid traces) and predicted *Z*-isomers (dashed traces) in different solvents with the absorbance normalized to 1 for the λ_{max} of the π-π* transition of the *E*-isomer.

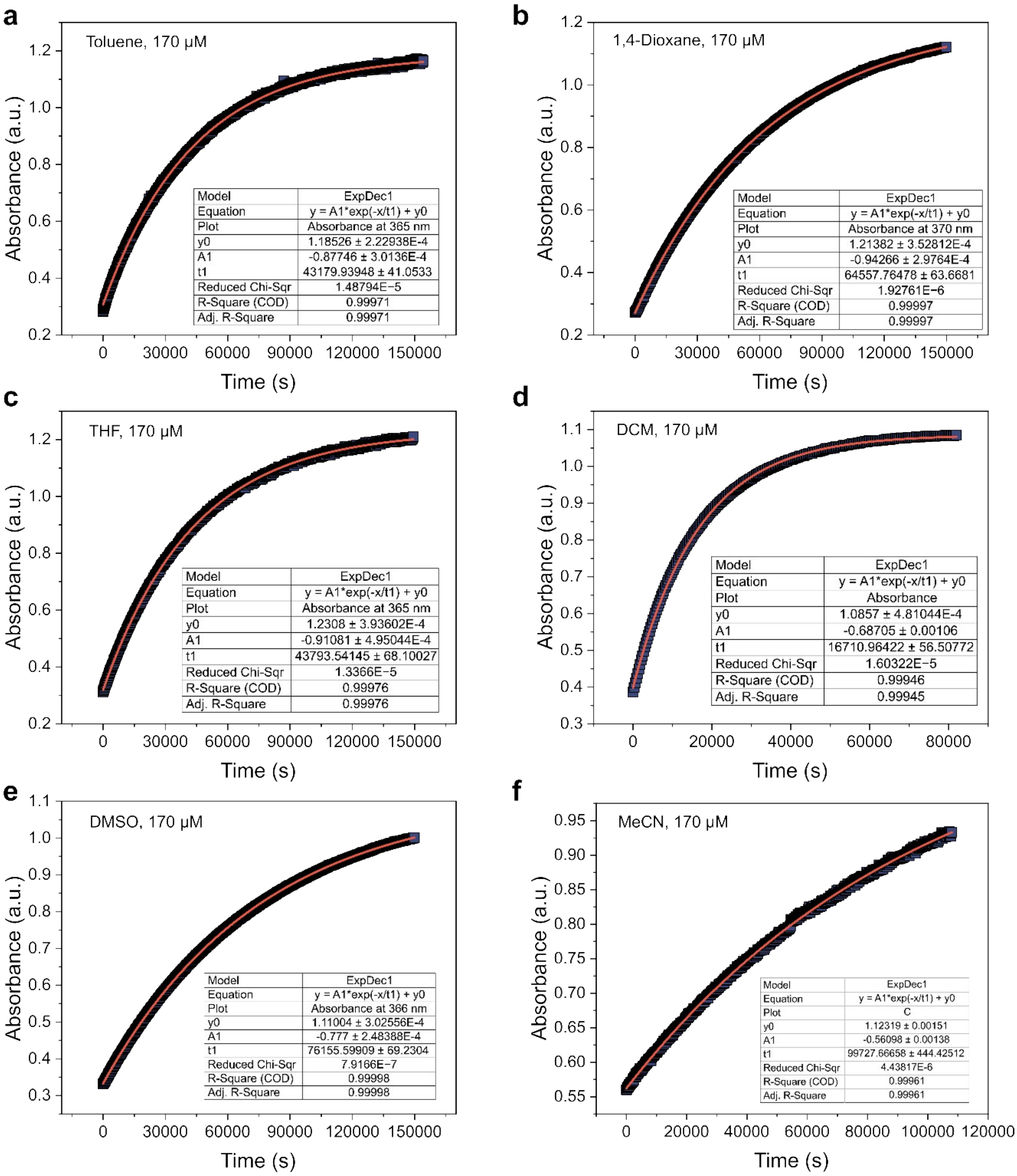

Figure S24. The change in absorbance of **4** (170 µM) at the λ_{max} of the *E*-isomer over time in different solvents at 20°C. The increase in absorbance relates to the thermal isomerization of the *Z*-isomer to the *E*-isomer. **a-e** were irradiated under 430 nm LED and **f** was irradiated under 365 nm LED for 5 minutes before the measurements started. The exponential fittings were applied to determine the rate constants and thus thermal half-lives.

5. Computational Studies

All underlying (TD-)DFT calculations were performed using the ORCA 5.0.4 software.[11–13] Other programs we used as part of our approach are CREST 2.12[14,15] and CENSO 1.2.1 for conformational search,[16] and NCIPLOT 4.0 for non-covalent interaction analysis.[17–19] Images of molecules were created with VMD 1.9.2.[20] An overview of the computational data of photoswitches **1-4** is presented in Figure S30-S33 below. An overview of the theoretical data is presented in Table S9.

Table S9. Summary of the theoretical data obtained for switches **1**-**4**.

Imine	Isomer	$\Delta G^{\ddagger}$ (kJ/mol)	$t_{1/2}$	$\Delta G_{Z\text{-}E}$ (kJ/mol)	E^{D4} (kJ/mol)
**1**	*E*	69.35	0.3 s	18.35	-150.63
	Z				-158.11
**2**	*E*	76.63	5.2 s	27.14	-140.28
	Z				-147.25
**3**	*E*	97.65	8.1 h	10.39	-216.99
	Z				-234.44
**4**	*E*	106.15	11 days	-2.45	-296.86
	Z				-307.34

5.1 Conformer Search and Geometry Optimisation of *E* and *Z* isomers

We began our studies by investigating the thermally accessible conformers of the imine switches at room temperature using the Conformer-Rotamer Ensemble Sampling Tool (CREST). Based on metadynamics[15] simulations and stepwise optimisation at semiempirical GFN2-xTB level,[21,22] this tool is suitable for finding low-energy conformers quickly and efficiently. The identified conformers were then refined using the Commandline Energetic Sorting (CENSO) algorithm. This process ranked the conformers based on their free energy at DFT-level of theory and optimised their geometries further using the meta-generalized-gradient approximation (mGGA) functional r^2SCAN-3c in combination with a def2-mTZVPP basis set.[23] The geometries of the lowest energy *E* and *Z* conformers were further optimised at ωB97X-D4/def2-TZVPP level of theory[24–29] with a CPCM solvation model for acetonitrile.[30] Ground-state equilibrium geometries of the *E* and *Z* isomers were confirmed by the absence of imaginary vibrational frequencies. The calculated properties shown in Table S9 in general match the experimental results very well, substantiating the used method. It is worth mentioning, however, that the computed $\Delta G_{Z\text{-}E}$ value for **4** implies that the *E* and *Z* isomers

are close to being in thermal equilibrium, slightly shifted towards *Z*. From the crystal structure of **4** shown in Figure 2d, and the ^{1}H NMR studies (Figure S14), however, it is clear that the *E*-isomer is indeed the thermodynamically stable isomer. This discrepancy highlights the limitations of the method used.

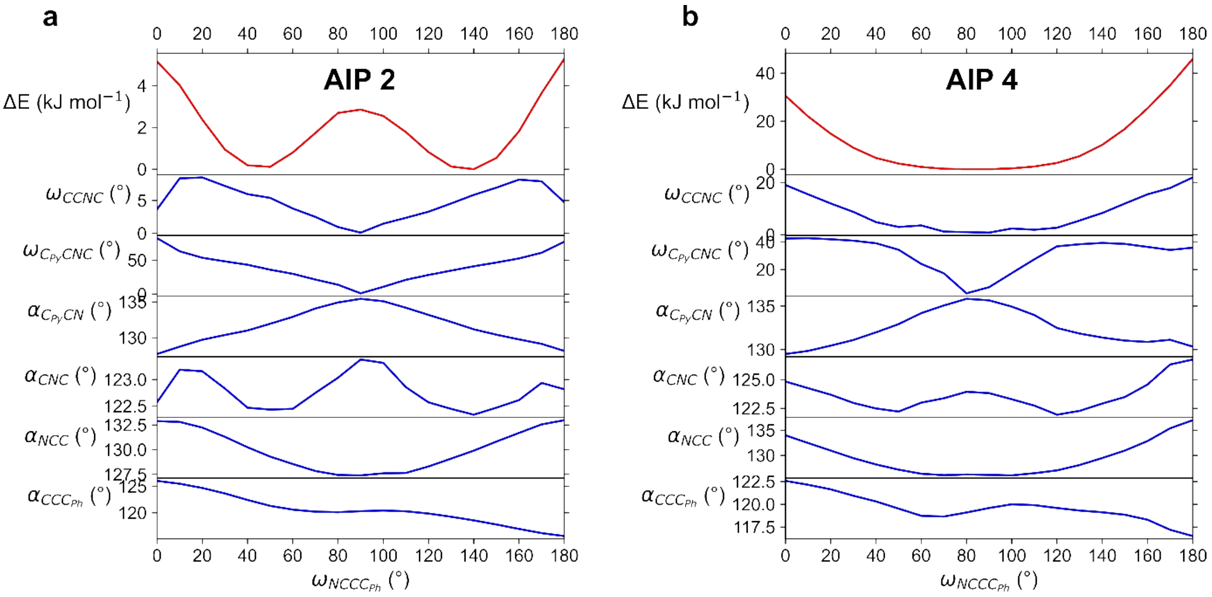

Figure S25. The scan of dihedral angle ω_{NCCCPh} from 0 to 180° in 10° increments, for **a**, AIP **2**, and **b**, AIP **4**, showing its effect on several structural parameters, including change in energy (shown in red).

Furthermore, to gauge the flexibility of the structures in their optimized state, specifically for the twisted vs T-shape structures of *Z*-**2** and *Z*-**4** respectively, we performed scans of the ω_{NCCCPh} angle (Figure S25). The scan of **2** indicates that the lowest energy conformation is that of a twisted geometry, and that the T-shape geometry is a local maximum in energy. It is notable that the lowest energy conformation of **4** is a T-shape.

5.2 Conformer Search and Geometry Optimisation of Transition States

To identify the type of transition state exhibited by these imine photoswitches, we performed Relaxed Surface Scans and Nudged Elastic Band (NEB) calculations.[31–33]

A representative example of a relaxed scan (ωB97X-D4/def2-TZVPP) is shown in Figure S26a, where the angles ω (CCNC dihedral angle) and α (CNC angle) are varied in 15° and 10° increments, respectively. In all cases studied, an inversion pathway was observed as the lowest energy pathway to convert *Z*-isomers to their *E*-form. Starting from the highest-energy structure of the inversion pathway, we were able to locate a transition state with linear C=N-C bonding (α ≈ 180°) upon optimisation. However, no transition state could be identified for the

rotation pathway, not even when broken symmetry (U)DFT was used, which is why we focused solely on the inversion pathway.

We were able to substantiate this observation by performing climbing-image NEB calculations. Due to the high computational cost of this method, we resorted to the mGGA functional r²SCAN-3c (def2-mTZVPP) after extensive testing of different methods (Figure S27). As an example, the minimum energy path of imine **2** is shown in Figure S26b, where 12 intermediate images were used. The climbing images marked in red shows an almost linear C=N-C bond in all cases and converged to the discussed inversion transition state upon optimization.

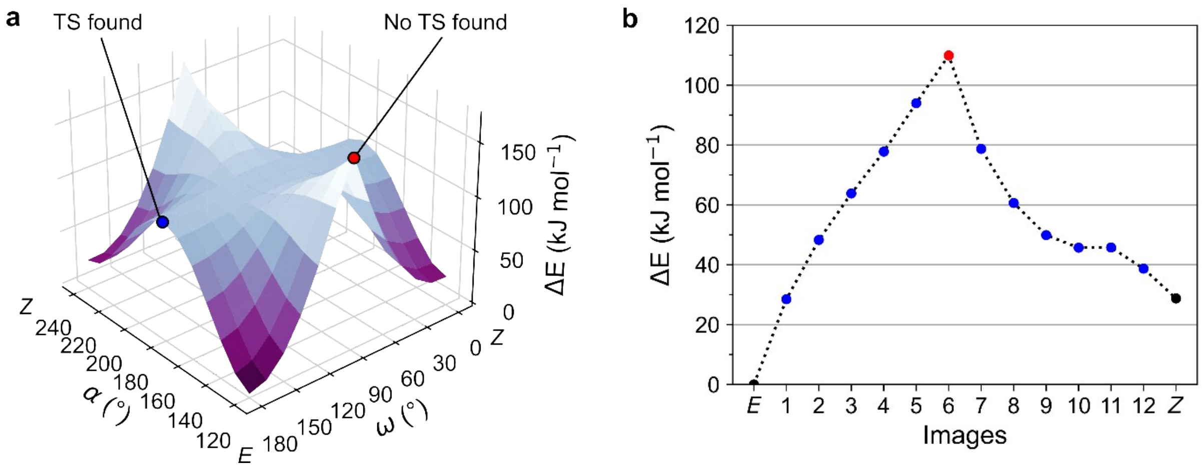

Figure S26. a, The PES of imine **2** as a function of scanned angles α and ω, obtained at ωB97X−D4/def2−TZVPP level of theory. Note that the TS corresponds to a linear geometry. **b**, The minimum energy path of the *E*/*Z* isomerism of **2** obtained from NEB calculation at r²SCAN−3c/def2−mTZVPP level of theory. The climbing image is highlighted in red.

Having identified transition state structures for all investigated structures, a conformational search of the transition state was applied using CREST. In this process, the TS mode was fixed (α = 180°) and conformers were searched as described above. As expected, a greater number of conformers were identified for imine switches with a greater number of flexible and rotatable appendages, for example **4**. The lowest-energy conformers were subsequently optimized at ωB97X-D4/def2-TZVPP level and the resulting transition state with the lowest energy was selected for the following calculations. All transition states were confirmed by the presence of a single imaginary vibrational frequency.

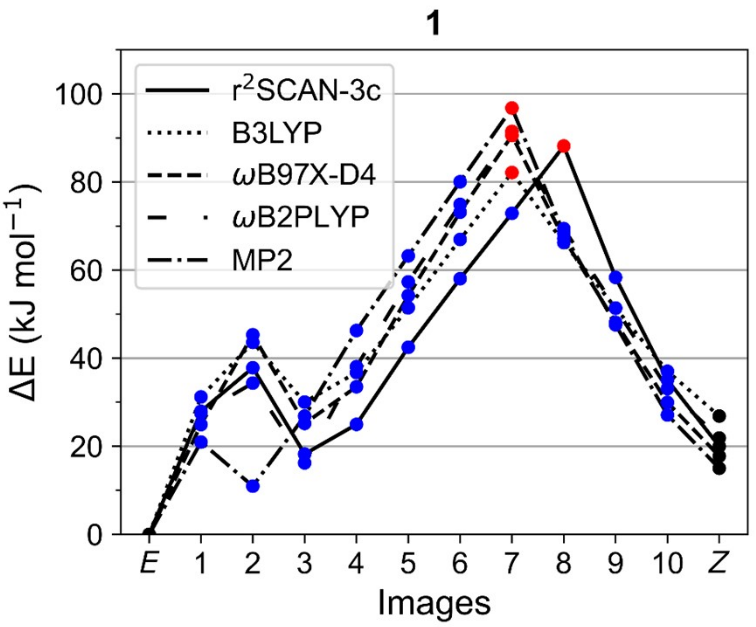

Figure S27. Minimum energy paths of the *E*/*Z* isomerization of **1**, obtained from NEB calculations using different methods. The respective climbing images are highlighted in red. All methods were combined with a def2−(m)TZVPP basis set.

5.3 Theoretical Investigation of Thermal Isomerisation Rates and Half-Lives

The rate of thermal isomerisation from the metastable *Z*-isomer to the thermodynamically stable *E*-isomer via the optimised transition state can be approximated using Eyring theory (Figure S28a).[6] The rate, $k_{Z\to E}$, of *Z* to *E* isomerisation is given by,

$$k_{Z\to E}(T) = \frac{k_B T}{h} \cdot e^{\frac{-\Delta G^{\ddagger}}{RT}} \tag{1}$$

where k_B is the Boltzmann constant, *T* is the temperature in Kelvin, *h* is the Planck constant, $\Delta G^{\ddagger}$ is the difference in the Gibbs energy of the *Z*-isomer and the transition state, and *R* is the gas constant. Once the rate is known, the thermal half-life, $t_{1/2}$, can be calculated from

$$t_{1/2} = \frac{ln2}{k_{Z\to E}} \tag{2}$$

Thermal half-lives predicted from quantum chemical calculations using this approach are able to reproduce the observed trends of experimental studies.[1–4] Whereas in some of these studies the half-life was calculated as a weighted average of different transition states, this approach did not yield any improvement in our case.

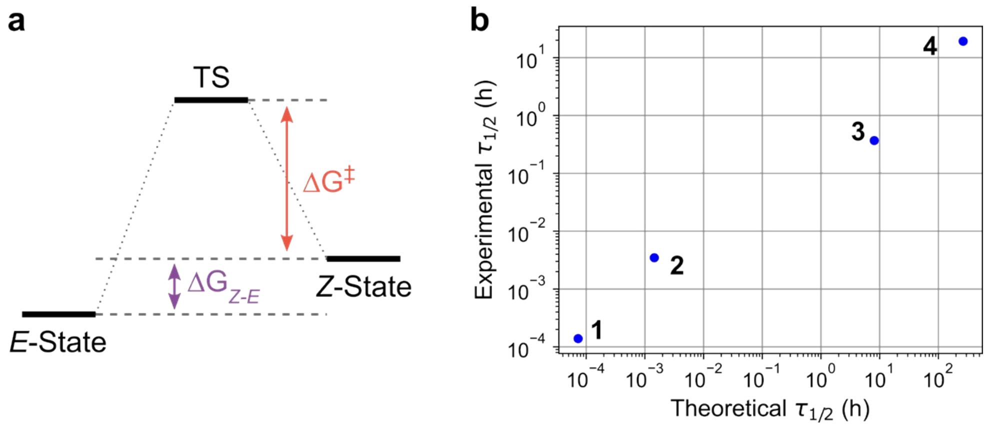

Figure S28. **a**, Schematic energy level diagram indicating how $\Delta G^{\ddagger}$ and $\Delta G_{Z\text{-}E}$ are calculated. **b**, The plot of the logarithm of the measured experimental thermal half-life against the logarithm of the theoretically determined thermal half-life at 20 °C.

5.4 Non-Covalent Interaction (NCI) Analysis

Non-covalent interaction analysis was used to rationalize observed differences in the geometries and thermal half-lives of the imine switches. This approach provides a visual representation of weak intramolecular interactions in the low-density regime. NCI analysis was performed using the NCIPLOT 4.0 program starting from the electron density of the optimized *E*, *Z* and TS structures obtained at ωB97X-D4/def2-TZVPP level.

5.5 Excited State Analysis

TD-DFT calculations were performed to investigate the photophysical properties of the imine switches. Here we used B3LYP/def2-TZVPP[34,35] to calculate the transition wavelengths and oscillator strengths, since this method provides the best agreement with the experimental spectra (Figure S29). We are aware that a range-separated hybrid functional such as ωB97X-D4 is conceptually more appropriate, but in our case underestimates the transition wavelengths significantly. We performed Natural Transition Orbital (NTO) analysis[36] for the associated lowest energy singlet transitions in order to help assignment of the experimental UV/vis absorption bands. The NTOs obtained with both functionals show no qualitative differences.

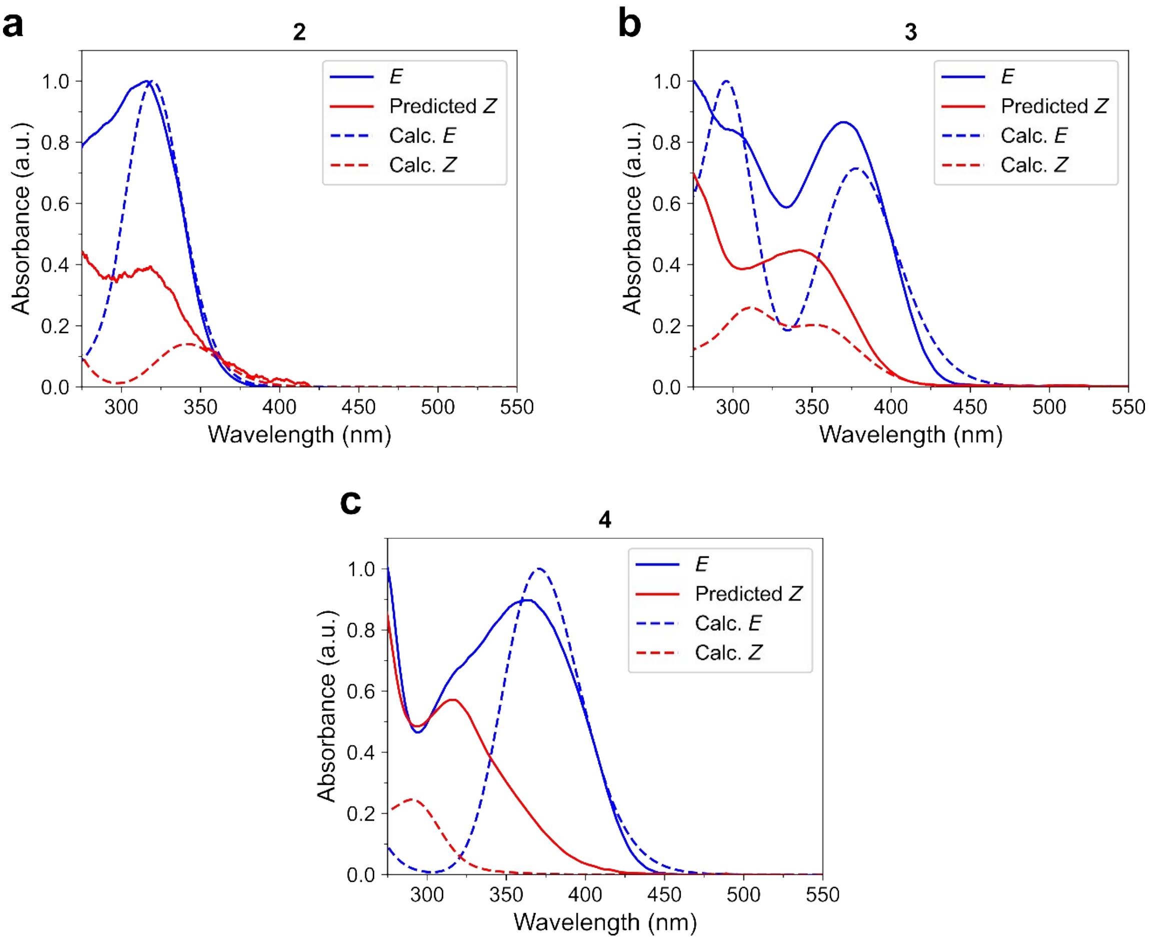

Figure S29. Plots of the theoretically calculated (Calc.) UV/vis absorption spectrum (dashed traces) along with the experimentally obtained UV/vis absorption spectrum (solid traces) for the *E* isomer, and predicted *Z* isomer (obtained from the procedure described in Section 4.5) of: **a**, AIP **2**; **b**, AIP **3**; **c**, AIP **4**. The negative photochromic behaviour that was observed experimentally for **3** and **4** is supported by these TDDFT calculations. The transition wavelengths and oscillator strengths obtained at the B3LYP/def2-TZVPP level were convoluted with a Gaussian broadening function (FWHM = 0.5 eV) and normalized to the highest peak.

5.6 Theoretical Data for Photoswitch 1

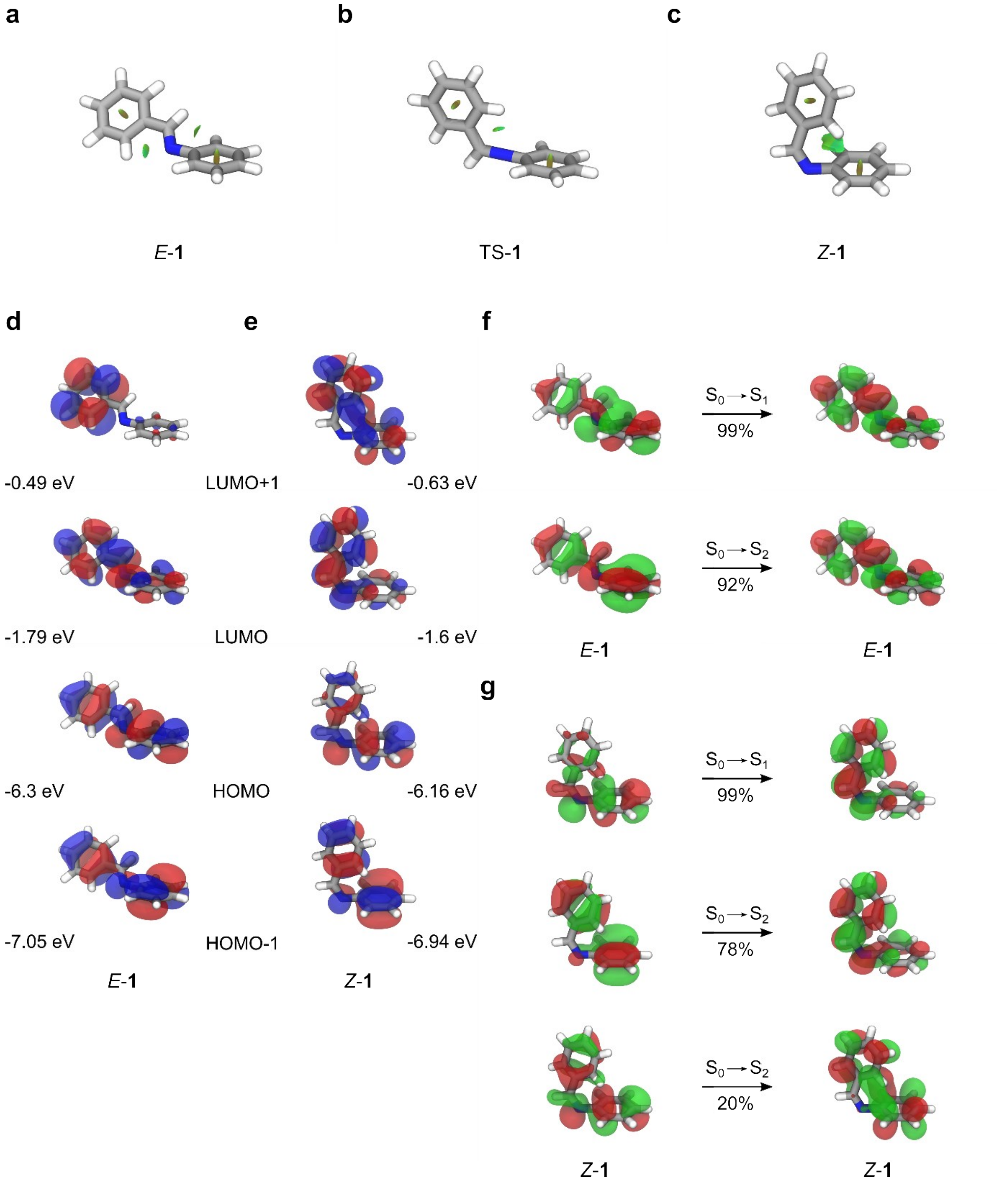

Figure S30. The geometry-optimised structures of **a**, *E*-**1**, **b**, TS-**1** and **c**, *Z*-**1** with NCI surfaces displayed. Frontier Molecular Orbitals (FMOs) of **d**, *E*-**1** and **e**, *Z*-**1** and corresponding energies. NTO pairs ("hole"→"particle") of the $S_0 \rightarrow S_1$ and $S_0 \rightarrow S_2$ transitions of **f**, *E*-**1** and **g**, *Z*-**1** with percentage contributions stated.

5.7 Theoretical Data for Photoswitch 2

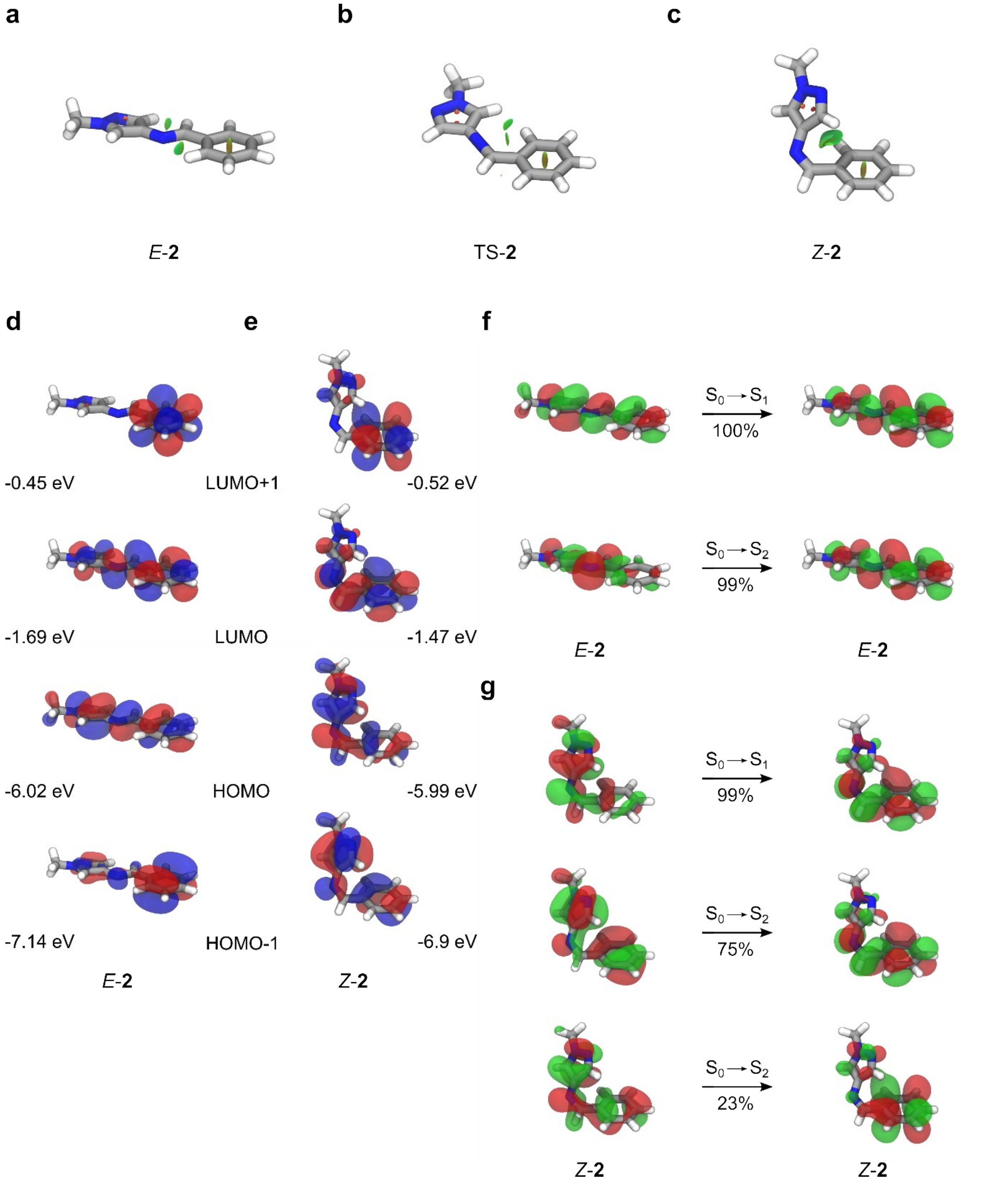

Figure S31. The geometry-optimised structures of **a**, *E*-**2**, **b**, TS-**2** and **c**, *Z*-**2** with NCI surfaces displayed. Frontier Molecular Orbitals (FMOs) of **d**, *E*-**2** and **e**, *Z*-**2** and corresponding energies. NTO pairs ("hole"→"particle") of the $S_0 \rightarrow S_1$ and $S_0 \rightarrow S_2$ transitions of **f**, *E*-**2** and **g**, *Z*-**2** with percentage contributions stated.

5.8 Theoretical Data for Photoswitch 3

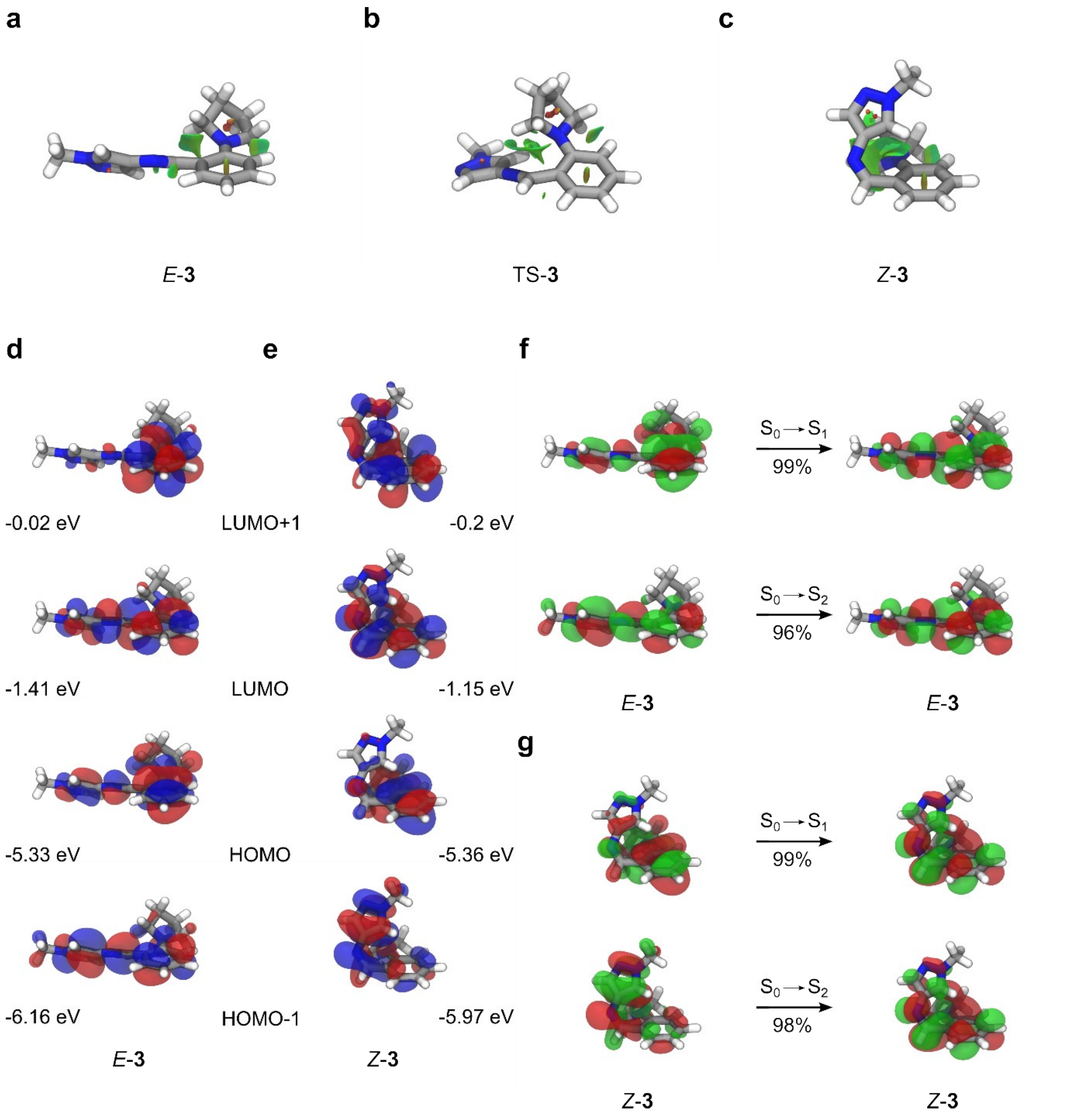

Figure S32. The geometry-optimised structures of **a**, *E*-**3**, **b**, TS-**3** and **c**, *Z*-**3** with NCI surfaces displayed. Frontier Molecular Orbitals (FMOs) of **d**, *E*-**3** and **e**, *Z*-**3** and corresponding energies. NTO pairs ("hole"→"particle") of the $S_0 \rightarrow S_1$ and $S_0 \rightarrow S_2$ transitions of **f**, *E*-**3** and **g**, *Z*-**3** with percentage contributions stated.

5.9 Theoretical Data for Photoswitch 4

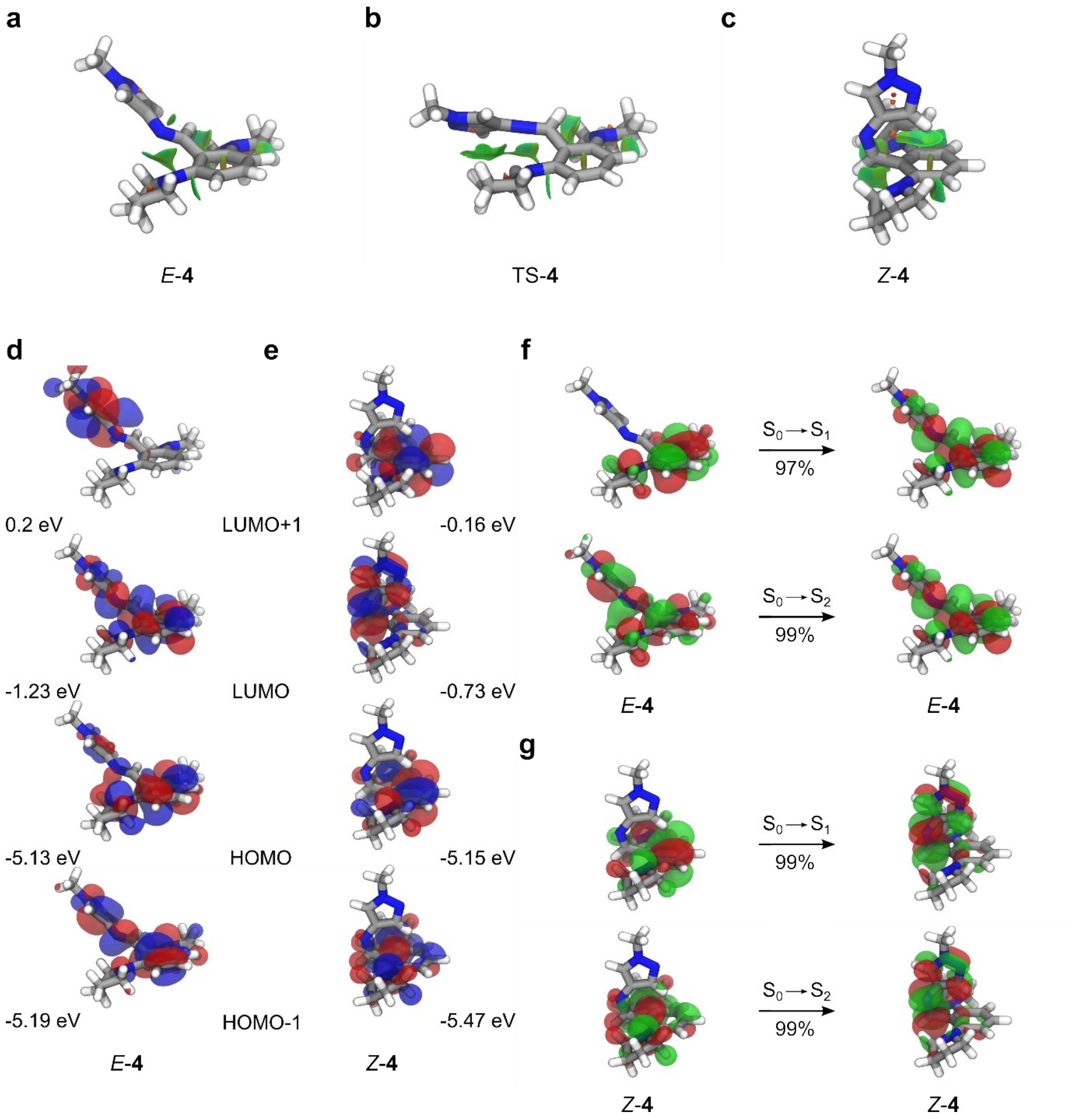

Figure S33. The geometry-optimised structures of **a**, *E*-**4**, **b**, TS-**4** and **c**, *Z*-**4** with NCI surfaces displayed. Frontier Molecular Orbitals (FMOs) of **d**, *E*-**4** and **e**, *Z*-**4** and corresponding energies. NTO pairs ("hole"→"particle") of the $S_0 \rightarrow S_1$ and $S_0 \rightarrow S_2$ transitions of **f**, *E*-**4** and **g**, *Z*-**4** with percentage contributions stated.

References

1 G. M. Sheldrick, *Acta Crystallogr. A*, 2015, **71**, 3–8.

2 Y. Luo, M. Utecht, J. Dokić, S. Korchak, H.-M. M. Vieth, R. Haag and P. Saalfrank, *ChemPhysChem*, 2011, **12**, 2311–2321.

3 T. Matsuhira, H. Yamamoto, A. Onoda, T. A. Okamura and N. Ueyama, *Org. Biomol. Chem.*, 2006, **4**, 1338–1342.

4 P. J. Coelho, M. C. R. Castro and M. M. M. Raposo, *J. Photochem. Photobiol. A Chem.*, 2013, **259**, 59–65.

5 V. Gold, Ed., *The IUPAC Compendium of Chemical Terminology*, International Union of Pure and Applied Chemistry (IUPAC), Research Triangle Park, NC, 2019.

6 H. Eyring, *J. Chem. Phys.*, 1935, **3**, 107–115.

7 E. Fischer, *J. Phys. Chem.*, 1967, **71**, 3704–3706.

8 *Proc. R. Soc. London. Ser. A. Math. Phys. Sci.*, 1956, **235**, 518–536.

9 K. Stranius and K. Börjesson, *Sci. Rep.*, 2017, **7**, 41145.

10 C. Reichardt, *Chem. Rev.*, 1994, **94**, 2319–2358.

11 F. Neese, *WIREs Comput. Mol. Sci.*, 2012, **2**, 73–78.

12 F. Neese, *WIREs Comput. Mol. Sci.*, 2018, **8**, e1327.

13 F. Neese, F. Wennmohs, U. Becker and C. Riplinger, *J. Chem. Phys.*, 2020, **152**, 224108.

14 P. Pracht, F. Bohle and S. Grimme, *Phys. Chem. Chem. Phys.*, 2020, **22**, 7169–7192.

15 S. Grimme, *J. Chem. Theory Comput.*, 2019, **15**, 2847–2862.

16 S. Grimme, F. Bohle, A. Hansen, P. Pracht, S. Spicher and M. Stahn, *J. Phys. Chem. A*, 2021, **125**, 4039–4054.

17 E. R. Johnson, S. Keinan, P. Mori-Sánchez, J. Contreras-García, A. J. Cohen and W. Yang, *J. Am. Chem. Soc.*, 2010, **132**, 6498–6506.

18 J. Contreras-García, E. R. Johnson, S. Keinan, R. Chaudret, J.-P. Piquemal, D. N. Beratan and W. Yang, *J. Chem. Theory Comput.*, 2011, **7**, 625–632.

19 R. A. Boto, F. Peccati, R. Laplaza, C. Quan, A. Carbone, J.-P. Piquemal, Y. Maday and J. Contreras-García, *J. Chem. Theory Comput.*, 2020, **16**, 4150–4158.

20 W. Humphrey, A. Dalke and K. Schulten, *J. Mol. Graph.*, 1996, **14**, 33–38.

21 C. Bannwarth, E. Caldeweyher, S. Ehlert, A. Hansen, P. Pracht, J. Seibert, S. Spicher and S. Grimme, *WIREs Comput. Mol. Sci.*, 2021, **11**, e1493.

22 S. Grimme, C. Bannwarth and P. Shushkov, *J. Chem. Theory Comput.*, 2017, **13**, 1989–2009.

23 S. Grimme, A. Hansen, S. Ehlert and J.-M. Mewes, *J. Chem. Phys.*, 2021, **154**, 064103.

24 A. Najibi and L. Goerigk, *J. Comput. Chem.*, 2020, **41**, 2562–2572.

25 E. Caldeweyher, C. Bannwarth and S. Grimme, *J. Chem. Phys.*, 2017, **147**, 034112.

26 E. Caldeweyher, S. Ehlert, A. Hansen, H. Neugebauer, S. Spicher, C. Bannwarth and S. Grimme, *J. Chem. Phys.*, 2019, **150**, 154122.

27 E. Caldeweyher, J.-M. Mewes, S. Ehlert and S. Grimme, *Phys. Chem. Chem. Phys.*, 2020, **22**, 8499–8512.

28 F. Weigend and R. Ahlrichs, *Phys. Chem. Chem. Phys.*, 2005, **7**, 3297.

29 F. Weigend, *Phys. Chem. Chem. Phys.*, 2006, **8**, 1057.

30 V. Barone and M. Cossi, *J. Phys. Chem. A*, 1998, **102**, 1995–2001.

31 G. Henkelman and H. Jónsson, *J. Chem. Phys.*, 2000, **113**, 9978–9985.

32 G. Mills, H. Jónsson and G. K. Schenter, *Surf. Sci.*, 1995, **324**, 305–337.

33 V. Ásgeirsson, B. O. Birgisson, R. Bjornsson, U. Becker, F. Neese, C. Riplinger and H. Jónsson, *J. Chem. Theory Comput.*, 2021, **17**, 4929–4945.

34 A. D. Becke, *J. Chem. Phys.*, 1993, **98**, 5648–5652.

35 P. J. Stephens, F. J. Devlin, C. F. Chabalowski and M. J. Frisch, *J. Phys. Chem.*, 1994, **98**, 11623–11627.

36 R. L. Martin, *J. Chem. Phys.*, 2003, **118**, 4775–4777.
